# RecFOR epistasis group: RecF and RecO have distinct localizations and functions in *Escherichia coli*

**DOI:** 10.1093/nar/gkz003

**Published:** 2019-01-18

**Authors:** Sarah S Henrikus, Camille Henry, Harshad Ghodke, Elizabeth A Wood, Neema Mbele, Roopashi Saxena, Upasana Basu, Antoine M van Oijen, Michael M Cox, Andrew Robinson

**Affiliations:** 1 Molecular Horizons Institute and School of Chemistry and Biomolecular Science, University of Wollongong, Wollongong, Australia; 2 Illawarra Health and Medical Research Institute, Wollongong, NSW 2500, Australia; 3 Department of Biochemistry, University of Wisconsin-Madison, WI 53706-1544, USA

## Abstract

In bacteria, genetic recombination is a major mechanism for DNA repair. The RecF, RecO and RecR proteins are proposed to initiate recombination by loading the RecA recombinase onto DNA. However, the biophysical mechanisms underlying this process remain poorly understood. Here, we used genetics and single-molecule fluorescence microscopy to investigate whether RecF and RecO function together, or separately, in live *Escherichia coli* cells. We identified conditions in which RecF and RecO functions are genetically separable. Single-molecule imaging revealed key differences in the spatiotemporal behaviours of RecF and RecO. RecF foci frequently colocalize with replisome markers. In response to DNA damage, colocalization increases and RecF dimerizes. The majority of RecF foci are dependent on RecR. Conversely, RecO foci occur infrequently, rarely colocalize with replisomes or RecF and are largely independent of RecR. In response to DNA damage, RecO foci appeared to spatially redistribute, occupying a region close to the cell membrane. These observations indicate that RecF and RecO have distinct functions in the DNA damage response. The observed localization of RecF to the replisome supports the notion that RecF helps to maintain active DNA replication in cells carrying DNA damage.

## INTRODUCTION

DNA damage and nucleotide depletion impede DNA replication and occasionally cause single-stranded gaps to be left in the wake of the replisome. These postreplicative gaps meet one of several fates: (i) gap filling by polymerases (1), (ii) homology-directed repair synthesis involving template switching (2–5) or (iii) conversion to potentially lethal double strand breaks that may be resolved by DNA recombination (4,6). In bacteria, the majority of postreplicative gaps are thought to be resolved by recombinational DNA repair via the RecFOR pathway (7,8).

The RecFOR pathway is mediated by the recombination mediator proteins—RecF, RecO and RecR. Their proposed function is to facilitate the loading of RecA onto single-stranded DNA (ssDNA) by displacing the ssDNA-binding protein, SSB (9–12). The *recF, recO* and *recR* genes form a putative epistasis group (5,13–21). This grouping is supported by several findings: (i) an identical level of increased sensitivity to UV irradiation when one of these functions is absent (22); (ii) almost identical deficiencies in DNA repair and recombination (23); (iii) the joint suppression of mutant alleles of all three genes by certain mutations in the *recA* gene (14,24); and (iv) the existence of a gene in bacteriophage λ that eliminates the requirement for all three genes in λ recombination (17,18). These observations have helped to perpetuate a misconception that the RecFOR pathway features a RecFOR complex (7,25). However, despite extensive examination, evidence for a RecFOR complex—even one formed transiently—is lacking.

The cohesiveness of a putative *recFOR* epistasis group begins to fray further upon closer examination of *in vivo* observations. First, many bacterial species lack a gene for RecF, but virtually all bacteria appear to have genes encoding RecR and one of two variants of RecO (25,26). Second, there are clear instances where the phenotype of a mutation in one of the *recFOR* genes diverges from the others (27–32). In *Bacillus subtilis*, RecF protein recruitment to repair foci is preceded by the appearance of RecO protein by several minutes (33). RecF is not essential, although its absence leads to a delayed increase in RecA foci formation when DNA is damaged (34).

The RecO and RecR proteins function together and are both necessary and sufficient for the nucleation of RecA on SSB-coated ssDNA *in vitro* (11,35). Further, RecO and RecR are essential for the formation of RecA foci *in vivo* (34). The RecO protein contains an oligonucleotide-binding fold (OB-fold) in its N-terminal domain and binds both ssDNA and double-stranded DNA (dsDNA) (36,37). In a RecA independent manner, RecO catalyses the annealing of complementary oligonucleotides and can also catalyse invasion of duplex DNA by a complementary ssDNA (37,38). The RecR protein has no known intrinsic enzymatic activities and exhibits poor functional conservation across bacteria. *Ec*RecR does not bind DNA, whereas the RecR homologs in *Deinococcus radiodurans* and *B. subtilis* both bind to DNA (39,40). In *Escherichia coli*, there is an apparent competition between RecF and RecO for RecR binding that may involve an interaction of both RecF and RecO with the C-terminal TOPRIM domain of RecR (41,42). RecR increases the apparent affinity of both RecO and RecF for DNA (11,43,44). Stimulation of RecA loading onto SSB-bound ssDNA does not occur in the presence of either RecO or RecR protein alone; it requires the formation of the RecOR complex (7,11,35). The RecOR-facilitated nucleation of RecA filaments onto SSB-coated ssDNA (RecAOR nucleation) is limited by access of RecOR to ssDNA, and requires an interaction of RecO with the C-terminus of SSB (45). The *Ec*RecR protein also forms a complex with RecF *in vitro* (11,43,44). As in the case of RecO, RecR increases the apparent affinity of RecF for DNA (11,43,44).

RecF is an SMC-like protein, exhibiting structural similarity with the head domain of the eukaryotic Rad50 protein, as well as sequence similarity to the head domains of the eukaryotic Structural Maintenance of Chromosomes (SMC) proteins (46). However, RecF lacks the long coiled-coil domains of Rad50. RecF belongs to the ATP-binding cassette (ABC) ATPase family of proteins, and it has the Walker A, Walker B and signature motifs characteristic of that family. ATP binding triggers RecF dimerization (46). The RecF protein (functioning in complex with RecR) cannot serve as a RecA loader (44). *In vitro*, RecFR binds randomly to dsDNA and can act as a barrier to RecA filament extension (44). RecF can also facilitate RecA filament extension on ssDNA by antagonizing the activity of the RecX inhibitor (47). Addition of RecF protein has a neutral or inhibitory effect on RecOR function (11,35,41,45,47), consistent with competition between RecF and RecO for RecR binding that may involve an interaction with the C-terminal TOPRIM domain of RecR (41,42). A RecF enhancement to RecOR-mediated loading has been observed when SSB is present in large excess (7). RecF can also have a positive effect on RecOR-mediated RecA loading when the interaction between RecO and SSB is abolished by utilizing an SSB mutant lacking the RecO interaction site in the SSB C-terminal tail (8). However, the latter two situations are unlikely to be physiologically relevant and the RecFR complex may well possess a function distinct from RecOR.

Given the complex and overlapping phenotypes, we set out to document the spatial and temporal behaviours of RecF and RecO proteins in live *E. coli* cells in response to DNA damage. Our observations provide insights into the intracellular localizations of RecF and RecO and reveal that the two proteins rarely interact with each other in cells during the DNA damage response.

## MATERIALS AND METHODS

### Strain construction

EAW670 is *E. coli* K-12 MG1655 *recF-YPet*. The 3′ end of the *recF* gene includes the promotor sequence for the *gyrB* gene downstream. We thus preserved the last 129 bp of *recF* and inserted an altered *recF* gene fused to sequences encoding *YPet* upstream (including mutant FRT-Kanamycin resistance-wt FRT cassette) using λ_RED_ recombineering. Positive colonies were selected for kanamycin resistance. The fusion gene *recF-YPet* encodes RecF, a C-terminal 12 amino acid spacer, followed by YPet. We similarly constructed EAW779, *E. coli* K-12 MG1655 *recF-mKate2*.

EAW814 is *E. coli* K-12 MG1655 *recO-YPet*. This construct was also made by λ_RED_ recombineering and contains a 3′ end duplication of *recO* gene (last 124 bp). This gene duplication is downstream of an altered *recO* gene fused to sequences encoding *YPet* (including mutant FRT-Kanamycin resistance-wt FRT cassette). EAW672 (*E. coli* K-12 MG1655 *recO-mKate2)* was constructed similarly.

EAW673 is *E. coli* K-12 MG1655 *recR-mKate2_(SL)_*. The fusion gene *recR-mKate2_(SL)_*encodes RecR, a C-terminal 11 amino acid spacer, followed by mKate2 (including mutant FRT-Kanamycin resistance-wt FRT cassette). This construct was also made by λ_RED_ recombineering and contains a 3′ end duplication of *recR* gene (last 247 bp). EAW897 (*E. coli* K-12 MG1655 *recR-mKate2_(LL)_)* and EAW898 (*E. coli* K-12 MG1655 *recR-YPet_(LL)_)* were constructed similarly except that they contain 20 amino acid spacers.

EAW642 is *E. coli* K-12 MG1655 *dnaX-mKate2*. The fusion gene *dnaX-mKate2* encodes DnaX, a C-terminal 11 amino acid spacer, followed by mKate2 (including mutant FRT-Kanamycin resistance-wt FRT cassette).

EAW676 (*recF-YPet recO-mKate2*) is a two-colour strain derived from EAW672 (*recO-mKate2*). The kanamycin resistance marker in EAW672 was removed via FLP-FRT recombination using the plasmid pLH29 (48) to obtain kanamycin sensitive EAW672. EAW676 was then constructed by replacing the *recF* gene of EAW672 with *recF-YPet*, a FRT-Kanamycin resistance-wt FRT cassette and the 3’ end duplication of *recF* using λ_RED_ recombineering. Colonies were selected for kanamycin resistance.

EAW762 (*recO-mKate2 dnaX-YPet*) is derived from the kanamycin sensitive parent strain EAW672 (*recO-mKate2*). To construct EAW762, λ_RED_ recombination was used to replace the *dnaX* gene of EAW672 with *dnaX-YPet* and a mutant FRT-kanamycin resistance-wt FRT cassette. Colonies were selected for kanamycin resistance. CJH0015 (*recF-mKate2 dnaX-YPet*) was constructed just as EAW762; the kanamycin sensitive EAW670 was infected with the P1 phage grown on JJC5945 (*dnaX-YPet*). We selected colonies for kanamycin resistance.

Deletion strains were constructed using λ_RED_ recombination, pKD46 was used for the λ_RED_ recombinase production and then removed from the strains (49). We created the following strains: EAW629 (Δ*recF*), EAW114 (Δ*recO*) and EAW669 (Δ*recR*). EAW788 was constructed using λ_RED_ recombination. We used pBLW24 (43) as a template to fuse the region encoding for *recF*(K36R) to the FRT-kanamycin resistance-wt FRT cassette. In all cases, deletion mutants and the *recF*(K36R) mutant maintain 3′ portions of each gene in order to preserve promoter sequences for genes downstream. Colonies were selected for kanamycin resistance. EAW214 (Δ*araBAD*) and HH020 (Δ*recA*) were used in previous studies (50,51).

Using λ_RED_ recombineering, we deleted *recF, recR* and *recA* in kanamycin sensitive EAW670 (*recF-YPet*). We produced EAW824 (*recF-YPet* Δ*recO*), SSH068 (*recF-YPet* Δ*recR*) and SSH070 (*recF-YPet* Δ*recA*). By analogy, deletion strains expressing RecO-mKate2 were constructed: EAW822 (*recO-mKate2* Δ*recF*), EAW697 (*recO-mKate2* Δ*recR*) and SSH067 (*recO-mKate2* Δ*recA*). We selected for kanamycin resistance.

To investigate the dependency of RecF on RecO, we created the two-colour strain EAW828 (*recO-mKate2 dnaX-YPet* Δ*recF*). The kanamycin sensitive parent strain EAW762 was transduced with a P1 phage lysate grown on EAW629. Colonies were selected for kanamycin resistance. EAW826 (*recF-mKate2 dnaX-YPet* Δ*recO*) was constructed in a similar manner, transducing CJH0015 with a P1 phage lysate grown on EAW114.

We further constructed a pair of two-colour strains (SSH114: *recF-mKate2 dnaX-YPet dnaB8*[Ts], SSH115: *recO-mKate2 dnaX-YPet dnaB8*[Ts]) that have a temperature-sensitive *dnaB* allele (52,53). The *dnaB8* allele encodes DnaB A130V (53). These strains were used to monitor the behaviours of RecF, DnaX and RecO under conditions where DNA replication is blocked (by shifting to the non-permissive temperature, 42°C) soon after inducing UV damage. SSH114 constructed by transducing the parent strain, CJH0015 (*recF-mKate2 dnaX-YPet dnaB*^+^), with a P1 phage lysate grown on WX31. Similarly, SSH115 was made by transducing EAW762 (*recO-mKate2 dnaX-YPet dnaB*^+^) with a P1 phage lysate grown on WX31. We also transduced the *dnaB8*(Ts) allele into MG1655 to produce HG362. HG362 was used to confirm the temperature sensitivity of all constructs in the MG1655 background (Supplementary Figure S14).

The two strains expressing either the fluorescent protein mKate2 (HG012) or YPet (HG013) were used to investigate if the fluorescent proteins themselves form foci after UV irradiation (Supplementary Figure S16). These two strains were produced by transforming either pBAD-Linker-mKate2 (for HG012) or pBAD-Linker-YPet (for HG013) into *E. coli* K-12 MG1655. The construction of these fluorescent proteins fused to a linker was previously published (54).

All constructs were confirmed by PCR and sequencing as required.

### Growth curves

Wild-type cells, deletion mutants and protein fusion constructs were grown in LB at 37°C in a microplate reader at a medium shaking rate (Biotek model Synergy2). Growth was monitored by measuring the optical density at a wavelength of 600 nm (OD_600_) over 10 h. For each strain, a biological quadruplet was recorded. To determine the growth of each strain, the average OD_600_ of the quadruplets and the corresponding standard deviation were plotted over time.

### Fitness of fusion strain constructs

Cell fitness was determined for each fusion strain using a modified growth competition assays described by Lenski *et al.* (56). In general, this two-colour colony assay is based on the colour difference of Ara^+^ and Ara^−^ colonies on tetrazolium arabinose indicator plates (TA plates). Ara^–^ colonies typically are red coloured, while Ara^+^ colonies are white. Ara^+^ and Ara^–^ cells can be counted and thus fitness in a mixed population of two strains can be assessed. Using this two-colour colony assay, the fitness of each fusion protein construct was measured in comparison to the parental strain that has the native gene instead of the fusion construct.

In preparation for the assay, individual overnight cultures of Ara^–^ and Ara^+^ cells were grown in 3 ml of LB at 37°C. The next day, a mixed culture of Ara^–^ and Ara^+^ cells was set up at a 1:1 ratio by volume. To start the experiment, 3 ml of medium was inoculated with 30 μl of the mixed culture and grown at 37°C. Fitness was assessed over the period of 72 h; cells were serial diluted in phosphate buffered saline (PBS) at 0, 24, 48 and 72 h. The dilutions were spread on plates containing TA plates and incubated at 37°C for 16 h before counting. We performed this assay competing Ara^+^ cells of each fusion protein construct with Ara^–^ cells of the corresponding parental strain and vice versa. We carried out triplicate measurements for each combination to determine the red and white percentage of the total population.

### UV survival assay

Cells were grown in LB overnight at 37°C. The next day, a 1/100 dilution of each culture was grown in LB medium (at 37°C, 150 rpm) until reaching mid-log phase (OD_600_ = 0.2). Cell cultures were then serial diluted in PBS by factors of ten down to 10^−5^ and 10 μl of each dilution was spotted in duplicates onto two LB plates. One of the plates was exposed to 60 J/m^2^ UV light using a cross-linker (Spectrolinker model XL1000 UV). The other was used as a no-exposure control. Unexposed and exposed plates were incubated at 37°C in the dark for 16 h. Images of plates were acquired with LAS4000 imager in digitalization mode (GE healthcare).

### SOS induction using mytomycin C

To investigate the levels of SOS induction in each fusion strain, we performed the β-galactosidase assay (Miller assay (57)) using a plasmid that expresses β-galactosidase from the SOS-inducible promoter for the *recN* gene (pEAW362) (58). Cells were grown in LB_Amp_ media (100 μg/ml ampicillin) overnight at 37°C and 150 rpm. The next day, a 1/100 dilution of the overnight cultures (total volume = 10 ml) was grown in LB_Amp_ medium (at 37°C, 150 rpm) until reaching an OD_600_ of 0.2 to 0.4. Two aliquots of 3 ml culture were taken. Mitomycin C was added to one 3 ml culture (to 0.2 μg/ml) and the other 3 ml culture was used as a control. The MMC-treated and untreated cells were grown for 2 h, then 1 ml of each culture was centrifuged and the pellet resuspended in Z buffer (0.06 M sodium phosphate dibasic heptahydrate, 0.04 M sodium phosphate monobasic, 0.01 M potassium chloride, 0.001 M magnesium sulfate, pH 7.0). Levels of SOS induction were determined by β-galactosidase assay (Miller) and were expressed as fold induction. Fold induction was determined by dividing the β-galactosidase activity of cells exposed to mitomycin C by the activity of the untreated cells.

### DNA damaging agent sensitivity assay

Cells were grown in LB overnight at 37°C. The next day, a 1/100 dilution of each culture was grown in LB medium (at 37°C, 150 rpm) until reaching mid log phase (OD_600_ = 0.2). Cell cultures were then serially diluted in PBS by factors of ten down to 10^−5^. Serial dilutions were spotted (spot volume 10 μl) on fresh LB plates and LB plates containing DNA damaging agent (which were protected from light). DNA damaging agents were added at the following concentrations: 5 μM nitrofurazone (NFZ), 3 μg/ml mitomycin C (MMC), 0.3 μM bleomycin, 0.1 μg/ml trimethoprim, 7.5 ng/ml ciprofloxacin or 5 mM hydroxyurea. Plates were incubated at 37°C for 16 h in the dark. Images of plates are acquired with LAS4000 imager in digitalization mode (GE healthcare).

### Temperature sensitivity assay

Cells were grown in LB overnight at 37°C. The next day, a 1/100 dilution of each culture was grown in LB medium (at 37°C, 150 rpm) until reaching mid log phase (OD_600_ = 0.2). Cell cultures were then serially diluted in PBS by factors of ten down to 10^−5^. Serial dilutions were spotted (spot volume 5 μl) on fresh LB plates. Plates were incubated at either 37 or 42°C for 16 h in the dark.

### SOS induction using DNA damaging agents

To compare the levels of SOS induction in deletion mutants with wild-type cells, we used cells that carry a vector for GFP expression from the SOS-inducible promoter of *recN* (pEAW903). Cells carrying the empty vector pET21A were used as a control. Cultures were grown in LB_Amp_ medium containing ampicillin at 37°C while shaking at 150 rpm until reaching mid log phase (OD_600_ = 0.2). For each strain, 200 μl of cultures were transferred into a 96-well microplate (Corning model black plate Costar). One culture was left untreated; the other culture was incubated with 0.5 μg/ml mitomycin C, 10 μM nitrofurazone, 0.4 μM bleomycin, 15 μg/ml trimethoprim, 10 ng/ml ciprofloxacin or 200 mM hydroxyurea. The 96-well microplate containing the untreated and treated cells was kept at 37°C for 10 h while medium shaking using a microplate reader (Biotek model Synergy2). The optical density (absorbance at 600 nm) and the fluorescence intensity (excitation, 485 nm – emission, 510 nm) were measured every 10 min. Cells carrying the empty vector and also untreated cells were expected to emit a low intensity fluorescence signal. Cells treated with DNA damaging agents that were carrying the SOS reporter plasmid were expected to emit a high intensity fluorescence signal due to the expression of GFP. For each strain and condition (treated or untreated), the expression level of the P*recN-GFP* was calculated at each time-point as followed. We divided the fluorescence signal gained from cells carrying the SOS reporter plasmid by their optical density and subtracted the fluorescence signal gained from cells carrying the empty vector by their optical density. We recorded triplicates for each condition. From these triplicates, two plots were generated. The average level of SOS induction and standard deviation were calculated and plotted as a function of time. The global SOS response over 10 h was illustrated as violin plots with identical max width using R software. Data are compiled from triplicate measurements. The median value is represented with a black dot along the vertical axis of each violin plot.

### Fluorescence microscopy

For all microscopy data, except for those comparing *dnaB* alleles and some controls (Supplementary Figures S16-17), wide-field fluorescence imaging was conducted on an inverted microscope (IX-81, Olympus with a 1.49 NA 100× objective) in an epifluorescence configuration. Continuous excitation was provided using semidiode lasers (Sapphire LP, Coherent) of the wavelength 514 nm (150 mW max. output) and 568 nm (200 mW max. output). RecF-mKate2 and RecO-mKate2 (CJH0015, EAW672, EAW676, EAW697, EAW762, EAW822, EAW826, EAW828, SSH067) were imaged using yellow excitation light (*λ* = 568 nm) at high intensity (2750 W cm^−2^ at EM gain 300), collecting emitted light between 610 and 680 nm (ET 645/75m filter, Chroma) on a 512 × 512 pixel EM-CCD camera (C9100–13, Hamamatsu). For RecF-YPet, RecO-YPet and DnaX-YPet imaging, we used green excitation (*λ* = 514 nm) at either lower (16 W cm^−2^ at EM gain 300) or higher laser power (160 W cm^−2^ at EM gain 300) for RecF-YPet and RecO-YPet strains (EAW670, EAW676, EAW814, EAW824, SSH068, SSH070) and 60 Wcm^−2^ for the DnaX-YPet strains (CJH0015, EAW762, EAW826, EAW828), collecting light emitted between 525 and 555 nm (ET540/30m filter, Chroma).

For the comparison of *dnaB* alleles, data were recorded on a Nikon Ti2-E microscope with a heated stage insert. Continuous excitation was provided by the same setup as described above. In all experiments including a temperature shift from 30 to 42°C, RecF-mKate2 and RecO-mKate2 (CJH0015, EAW762, SSH114, SSH115) were also imaged using yellow excitation light (*λ* = 568 nm) at high intensity (2750 W cm^−2^ at EM gain 100), collecting emitted light between 610 and 680 nm, (ET654/75m filter, Chroma) on a 512 × 512 pixel EM-CCD camera (C9100–13, Hamamatsu). DnaX-YPet (CJH0015, EAW762, SSH114, SSH115) was imaged using green excitation (*λ* = 514 nm) at lower (60 W cm^−2^ at EM gain 255), collecting light emitted between 525 and 555 nm (ET540/30m filter mounted in Nikon Ti2 Filter Cubes, Chroma).

Burst acquisitions (movies of 300 × 34 ms frames, continuous excitation with 514 nm light) were collected to characterize the motions of RecF-YPet and RecO-YPet molecules, and to determine the number of RecF-YPet and RecO-YPet molecules per cell. Single-colour time-lapse movies were recorded to visualize RecF-YPet or RecO-mKate2 binding to DNA (EAW670, EAW672, EAW697, EAW779, EAW814, EAW822, EAW824, SSH067, SSH068, SSH070). A set of two-images was recorded at an interval of 10 min for 3 h, UV irradiation just after the first image was taken (bright-field [34-ms exposure], YPet fluorescence [100-ms exposure] or bright-field [34-ms exposure], mKate2 fluorescence [100-ms exposure]). Two-colour time-lapse movies were recorded to visualize if RecF-YPet and RecO-mKate2 (EAW676) bind to DNA as a complex. Sets of three images were recorded (bright-field [34-ms exposure], mKate2 fluorescence [100-ms exposure], YPet fluorescence [100-ms exposure]) at an interval of 10 min for 3 h. To measure colocalization between RecF-mKate2 and RecO-mKate2 with the replisome marker (CJH0015, EAW762, EAW826, EAW828, SSH114, SSH115), we recorded time-lapse movies at the same intervals but different exposures for the replisome marker (bright-field [34-ms exposure], mKate2 fluorescence [100-ms exposure], YPet fluorescence [500-ms exposure]). All images were analysed with ImageJ (59). Example datasets have been made freely accessible (doi: 10.6084/m9.figshare.7409822).

### Flow cell designs

All imaging experiments were carried out in home-built quartz-based flow cells (62). These flow cells were assembled from a no. 1.5 coverslip (Marienfeld, REF 0102222), a quartz top piece (45 × 20 × 1 mm) and PE-60 tubing (Instech Laboratories, Inc.). Prior to flow-cell assembly, coverslips were silanized with aminopropyltriethoxy silane (Alfa Aeser). First, coverslips were sonicated for 30 min in a 5 M KOH solution to clean and activate the surface. The cleaned coverslips were rinsed thoroughly with MilliQ water and then treated with a 5% (v/v) solution of amino-propyl-triethoxysilane (APTES) in MilliQ water. The coverslips were subsequently rinsed with ethanol and sonicated in ethanol for 20 s. Afterward, the coverslips were rinsed with MilliQ water and dried in a jet of N_2_. Silanized slides were stored under vacuum prior to use.

To assemble each flow cell, polyethylene tubing (BTPE-60, Instech Laboratories, Inc.) was glued (BONDiT B-482, Reltek LLC) into two holes that were drilled into a quartz piece. After the glue solidified overnight, double-sided adhesive tape was stuck on two opposite sides of the quartz piece to create a channel. Then, the quartz piece was stuck to an APTES-treated coverslip. The edges were sealed with epoxy glue (5 Minute Epoxy, PARFIX). Each flow cell was stored in a desiccator under mild vacuum while the glue dried. Typical channel dimensions were 45 mm × 5 mm × 0.1 mm (length × width × height).

### Imaging in flow cells

For imaging experiments conducted at 37°C, cells were grown at 37°C in EZ rich defined medium (Teknova) that contained 0.2% (w/v) glucose (62). All strains that have a *kanR* cassette were grown in the presence of kanamycin (20 μg/ml). Cells were loaded into flow cells, allowed a few minutes to associate with the APTES surface, then loosely associated cells were removed by pulling through fresh medium. The experiment was then initiated by irradiating cells *in situ* with 254 nm UV light from a mercury lamp (UVP) at a fluence of 10 J m^−2^. Throughout the experiment, medium was pulled through the flow cell using a syringe pump, at a rate of 50 μl/min.

For imaging experiments conducted at the *dnaB8*(Ts) non-permissive temperature, cells were grown at 30°C in EZ rich defined medium (Teknova) that contained 0.2% (w/v) glucose (62). All strains have a *kanR* cassette, and thus, were grown in the presence of kanamycin (20 μg/ml). Cells were loaded into flow cells as described above at 30°C. Following acquisition of data at the first time-point (*t* = 0 min), the temperature was rapidly ramped up to 42°C. After 3 min, the stage reached a temperature of 39 to 41°C. Following this, cells were irradiated *in situ* with a brief pulse of 254 nm light (10 J m^−2^) through a quartz window in the flow cell. The temperature of the stage stabilized at 42°C within 5 min following the first acquisition, and was maintained constant at this value for the rest of the experimental time line. Throughout the experiment, medium was pulled through the flow cell using a syringe pump at a rate of 50 μl/min.

### Analysis of cell filamentation, RecF and RecO levels and foci per cell

We selected single cells to obtain information about RecF and RecO levels upon UV irradiation (>100 cells for every time-point). MicrobeTracker 0.937 (60), a MATLAB script, was used to create cell outlines as regions of interest (ROI). We manually curated cell outlines designated by MicrobeTracker before UV irradiation and at intervals of 30 min up to 120 min after UV irradiation. By obtaining cell outlines manually, we ensure accuracy and purely select non-overlapping, in-focus cells for analysis. These ROI were imported in ImageJ 1.50i. The cell outlines were then used to measure mean cell intensities, cell lengths and the number of foci per cell. Parameters describing foci (number, positions and intensities) were obtained using a Peak Fitter plug-in, described previously (61,62).

### Analysis of colocalization events

It has been shown that freely moving molecules diffuse quickly (*D* ≈ 10 μm^2^/s), whereas, DNA-bound molecules diffuse much slower (*D* ≈ 10^−5^ μm^2^/s) (63,64). The imaging conditions (34- or 100-ms exposures) used here separate freely diffusing molecules from bound molecules due to the difference in their diffusion behaviour; a focus represents a DNA bound molecule, and diffusive molecules increase the background signal.

Foci were classed as colocalized if their centroid positions (determined using our peak fitter tool) fell within 2.18 px (218 nm) of each other. The script used to quantify these data has been made freely available (doi: 10.6084/m9.figshare.7409822). We determined that for RecF-mKate2–DnaX-Pet localization, the background of RecF foci expected to colocalize with replisomes purely by chance is ∼4% when imaging at 37°C. This was calculated by taking the area of each cell occupied by replisome foci (including the colocalization search radius) and dividing by the total area of the cell. The value of 4% corresponds to the mean of measurements made over >200 cells. Since the foci density of replisomes stays fairly constant after UV irradiation, the chance colocalization of RecF-mKate2 foci with DnaX-YPet is ∼4%. Similarly, the chance colocalization of RecO-mKate2 with DnaX-YPet is ∼4% before and after UV irradiation. Similarly, chance colocalization is ∼4% for RecF with DnaX and RecO with DnaX in *dnaB8*(Ts) and *dnaB*^+^ at 30°C. After UV irradiation, at 42°C, in *dnaB*^+^, chance colocalization of RecF with DnaX and RecO with DnaX is also ∼4%. In contrast, chance colocalization of RecF with DnaX and RecO with DnaX decreases in *dnaB8*(Ts) at the non-permissive temperature (42°C) after UV irradiation. Chance colocalization is ∼0.5% at 90 min.

At 37°C, in *dnaB*^+^ cells, the chance colocalization of DnaX-YPet with RecF-mKate2 is similar to chance colocalization with replisomes due to a similar foci density before and after UV irradiation (chance colocalization ∼4%, >100 cells). The chance colocalization of RecO-mKate2 with RecF-YPet is ∼4% following UV irradiation. At 30°C, in *dnaB*^+^ and *dnaB8*(Ts), chance colocalization of DnaX-YPet with RecF-mKate2 is ∼2% because half the number of RecF foci per cell are detected. In *dnaB*^+^, chance colocalization is also ∼2% after UV irradiation at 42°C. In *dnaB8*(Ts), chance colocalization however drops after UV irradiation at the non-permissive temperature as the number of RecF foci per cell declines. At 90 min, chance colocalization is ∼0.5%.

In *dnaB*^+^ and *dnaB8*(Ts) under all conditions, there are <0.3 RecO foci per cell before UV irradiation, thus there is close to zero chance that a replisome focus or RecF focus will colocalize with a RecO focus by chance. At 30 min, in *dnaB*^+^ at 37 and 42°C, chance colocalization is expected to be <1% and at 120 min, the chance for colocalization is 1%. In *dnaB8*(Ts), chance colocalization is close to zero when imaging at 30°C as well as after UV irradiation at the non-permissive temperature because <0.3 foci per cell are detected.

### Analysis of RecF and RecO copy numbers per cell

The number of RecF-YPet and RecO-YPet molecules and thus the physiological concentration of RecF and RecO are extracted from the integrated fluorescence signal under each cell outline during burst acquisition experiments. Each cell exhibits an intensity decay which is composed of YPet bleaching, cellular auto-fluorescence and background fluorescence (62). Exciting with a higher laser power (160 W cm^−2^), *E. coli* MG1655 cells, expressing no YPet, exhibit auto-fluorescence equivalent to ∼2.5 YPet molecules which we corrected for. The background fluorescence was negligible (equivalent to <1 YPet molecule). After correcting for auto-fluorescence, the integrated fluorescence signal under each cell outline corresponds to the fluorescence signal of intracellular YPet molecules.

Images were corrected for the electronic offset and flattened to correct for inhomogeneity of the excitation beam (inhomogeneity was small at a laser power of 160 W cm^−2^; the brightest part at the centre of the image was 12% more intense than at the corners). For each cell, the mean YPet signal per pixel of the first frame from the time series experiments was extracted. The mean YPet signal multiplied by the cell area gives the integrated YPet intensity, which was used to determine the number of YPet molecules per cell.

The mean intensity of individual YPet molecules was determined by analysing single-molecule return events (Supplementary Figure S5), as previously described (62). For each cell, the number of RecF-YPet or RecO-YPet molecules was then calculated by dividing the mean YPet signal of the first frame from the burst acquisition experiments by the mean single-molecule intensity. The cellular concentration was calculated using the cell volume of each cell, determined during cell outline assignation in MicrobeTracker.

### Autocorrelation analysis and simulation of intensity versus time trajectories

Within the rapid acquisition movies, intensity fluctuations within regions of cells corresponding to RecF or RecO foci were monitored as a function of time. The resulting intensity versus time trajectories contain information on the binding and dissociation behaviours of RecF and RecO, convoluted with photobleaching effects (which cause an exponential loss of signal as a function of time) and noise (which by definition is not correlated in time). To gain information on the binding and dissociation behaviours of RecF and RecO, we calculated the autocorrelation functions for all trajectories recorded and determined the mean autocorrelation for each particular set of conditions. The averaged autocorrelation function contained three major components. Fast decorrelation occurred on the time scale of the integration time due to noise and transient binding event (*τ*_s_ < 0.034 s). The exponential decay in the autocorrelation curve was fitted starting from lag time 0.034 s (after the initial fast decorrelation) with single and double exponential-decay functions. Two major component timescales were present in the remainder of the autocorrelation curve (*τ*_m_ = 0.3 s for RecF-YPet and RecO-YPet, *τ*_l_ = 1.5 s for RecF-YPet and 2.2 s for RecO-YPet, Supplementary Figure S4). The amplitude of each component (*a*_s_, *a*_m_, *a*_l_) represents the weight for each autocorrelation components. Error bars for *a*_s_ were derived from the standard deviation of the error mean for each average autocorrelation function at lag time 0.034 s. Error bars for *a*_m_ and *a*_l_ were derived from the fit error.

An increase in signal intensity within foci, such as that observed upon dimerization of RecF, will cause an increase in the signal-to-noise ratio within trajectories. When the autocorrelation curve is determined, this will manifest as a reduction in the fast-decorrelating component. To determine what effect a 2-fold increase in focus intensity would have during autocorrelation analysis, we produced simulated trajectories in which complexes containing either one or two fluorescent molecules photobleached, bound to DNA, and dissociated from DNA. Simulations were run in Matlab 2012a using custom-written code (Appendix PDF). The simulator is comprised of three sub-routines. In the first sub-routine, binding/dissociation trajectories are generated for a complex (representing RecF or RecF_2_ binding to DNA). When bound, the complex produces signal (*I* = 1). When unbound, it produces none (*I* = 0). Binding and dissociation times are determined by randomly sampling user-defined distributions of *k*_on_ and*k*_off_. In the second sub-routine, similar trajectories are produced for individual molecules binding to each complex. In the third sub-routine, photobleaching trajectories are produced for each molecule in the simulation, by drawing randomly from a user-defined distribution of bleaching rates *τ*_bleach_. The three signals are then combined such that a molecule only produces signal when it is bound to the complex, the complex is bound to DNA and the molecule has not yet photobleached. Poissonian noise is added to the signal for each molecule according to a user-defined signal-to-noise parameter. Averaging is used to appropriately reduce noise when multiple molecules are bound. The key input parameters for simulation are: *N*_mol/comp_, the maximum number of molecules that can bind to each complex; *k*_on_(complex), the on-rate for complex binding to DNA; *k*_off_(complex), off-rate for complex dissociation from DNA; *k*_on_(molecule), on-rate for molecule binding to complex; *k*_off_(molecule), off-rate for molecules dissociating from complex; *τ*_bleach_, the mean photobleaching rate for molecules. Using this code, simulations were run for complexes that permanently contained either one or two molecules (of RecF), keeping all other parameters constant. The autocorrelation functions for one-molecule and two-molecule trajectories were compared.

## RESULTS

### *recF* and *recO* mutant phenotypes diverge depending upon DNA damaging agent

The *recF* and *recO* genes (along with *recR*) have been grouped to reflect the very similar phenotypes displayed by mutants lacking the function of the encoded proteins. We set out to systematically investigate the phenotype of mutations in these two genes, exploring a range of DNA damaging agents with different modes of action. To generate DNA damage, we treated cell cultures separately with NFZ, MMC, bleomycin (bleo), ciprofloxacin (cipro), hydroxyurea (HU) and trimethoprim (TMP). We did not further explore the effects of ultraviolet light exposure, as the original observation of phenotypic equivalence with this stressor (22) has been repeatedly reproduced in our laboratories and many others. The sensitivity of the mutant strains EAW629 (Δ*recF*), EAW114 (Δ*recO*), EAW669 (Δ*recR*) and EAW788 (*recF*[K36R]) to each DNA damaging agent was tested using a spot plate dilution assay. The mutants were compared to the wild-type strain MG1655 (wild-type), which is the genetic background into which all gene mutations were introduced.

**Figure 1:**
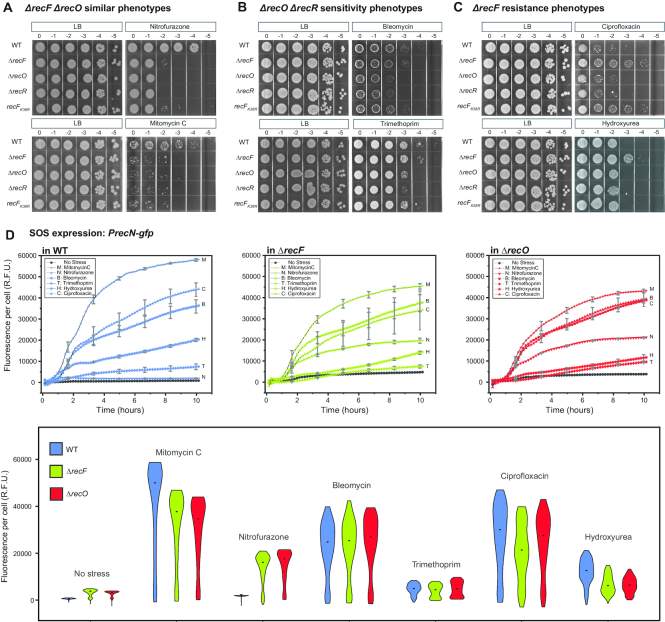
Cells lacking *recF* and *recO* present differences in sensitivity to DNA damaging agents. (**A**), (**B**) and (**C**) spot plate dilution assays of MG1655 (wild-type), EAW629 (Δ*recF*), EAW114 (Δ*recO*), EAW669 (Δ*recR*), EAW788 (*recF*[K36R]). Cells grown to exponential phase (OD_600_ ∼ 0.2) were serial diluted to the dilution 10^−5^. Serial dilutions were spotted on LB agar and LB agar supplemented with the indicated DNA damaging agent. Plates were incubated overnight at 37°C. Images show a representative experiment of independent triplicates. (**A**) Sensitivity of cells exposed to 5 μM NFZ or 3 μg/ml MMC. The sensitivities to NFZ and MMC are almost identical for Δ*recF*, Δ*recO*, Δ*recR* and *recF*(K36R) strains (Δ*recF* and *recF*(K36R) are slightly more resistant than Δ*recO*, Δ*recR* to NFZ). (**B**) Sensitivity of cells exposed to 0.3 μM bleo or 0.10 μg/ml TMP. Δ*recO*, Δ*recR* are ∼10 times more sensitive to bleo in comparison to wild-type, Δ*recF* and *recF*(K36R) mutants. (C) Sensitivity of cells exposed to 7.5 ng/ml cipro or 5 mM HU. Deletion of *recF* confers resistance to cipro and HU. The ATPase deficient *recF* mutant (*recF*[K36R]) confers resistance to cipro. (**D**) Expression of the SOS reporter fusion *PrecN-gfp* over a period of 10 h in wild-type (blue), Δ*recF* (green) and Δ*recO* strains (red). Cells grown to exponential phase (OD_600_ ∼ 0.2) were exposed to 10 μM NFZ (downward facing triangle), 0.5 μg/ml MMC (star-shaped), 0.4 μM bleo (square), 15 μg/ml TMP (diamond), 10 ng/ml cip (pentagon) or 200 mM HU (upward facing triangle). Untreated cells (grey circle) were used as a control. The expression of *PrecN-gfp* per cell is expressed in relative fluorescent units (R.F.U). Upper three panels show the *PrecN-gfp* average expression as function of time for wt (left, blue), Δ*recF* (middle, green) and Δ*recO* (right, red). Error bars represent the standard deviation of biological triplicates. Lower panel, violin plot representing the global expression of *PrecN-gfp*, the central dot indicates the median value.

In these trials, three patterns emerged. First, in some cases, there was no evident difference between the Δ*recF* and Δ*recO* phenotype, congruent with previous reports on UV-induced damage. When cells were challenged with NFZ or MMC, the strains carrying deletions in any of the three genes displayed an approximately equal degree of sensitivity (Figure 1A). In the second pattern, Δ*recO* produced results that diverged from wild-type, while Δ*recF* did not. When cells were exposed to bleomycin or trimethoprim, the strains Δ*recO* and Δ*recR* were ∼10-fold more sensitive than the wild-type cells or a strain lacking *recF* (Figure 1B). Third and finally, strains with a *recF* deletion uniquely diverged from the wild-type phenotype in some cases. When cells were exposed to ciprofloxacin or HU, the Δ*recF* strain was more resistant (up to 2 logs for ciprofloxacin and ∼1 log for HU, depending on the concentration of stressor) (Figure 1C). We also investigated the contribution of the RecF ATPase activity to the *recF* phenotype, using the RecF ATPase deficient mutant (*recF*[K36R]). Interestingly, cells with the *recF*(K36R) mutation were more resistant to ciprofloxacin but not to HU (Figure 1C). Altogether, the results reveal several DNA damaging conditions in which the Δ*recO* and Δ*recF* mutations produce quite different phenotypes.

We set out to determine if the difference between the Δ*recF* and Δ*recO* phenotypes to the different DNA damaging agents was also reflected in a difference in SOS induction. We used a plasmid expressing GFP from the SOS-inducible promoter for the *recN* gene, pEAW903 (p*PrecN-gfp*) (65). Deletion strains of *recF* and *recO* carrying p*PrecN-gfp* were grown to exponential phase and treated with the various DNA damaging agents (NFZ, MMC, bleo, cipro, HU or TMP). We then monitored GFP expression for 10 h. Exposure to NFZ induced little or no *PrecN-gfp* expression in the wild-type cells and moderate expression (∼18 000 R.F.U.) in Δ*recF* and Δ*recO* cells (Figure 1D). Exposure to bleo, cipro or TMP triggered similar SOS induction profiles for all three strains (∼40 000 R.F.U with bleo or cipro and ∼12 000 R.F.U with TMP). Exposure to HU or MMC showed a slight reduction in *PrecN-gfp* expression in Δ*recF* or Δ*recO* mutants relative to wild-type cells (∼12 000 R.F.U versus ∼20 000 R.F.U. for HU; ∼45 000 R.F.U versus ∼58 000 R.F.U for MMC). Overall, we found differences in *recF* and *recO* phenotypes suggesting that RecF and RecO might have distinct functions. We thus chose to further investigate RecF and RecO behaviour on the single-molecule level in live *E. coli* cells.

### RecF and RecO have different DNA binding behaviours and respond differently to UV irradiation

**Figure 2.**
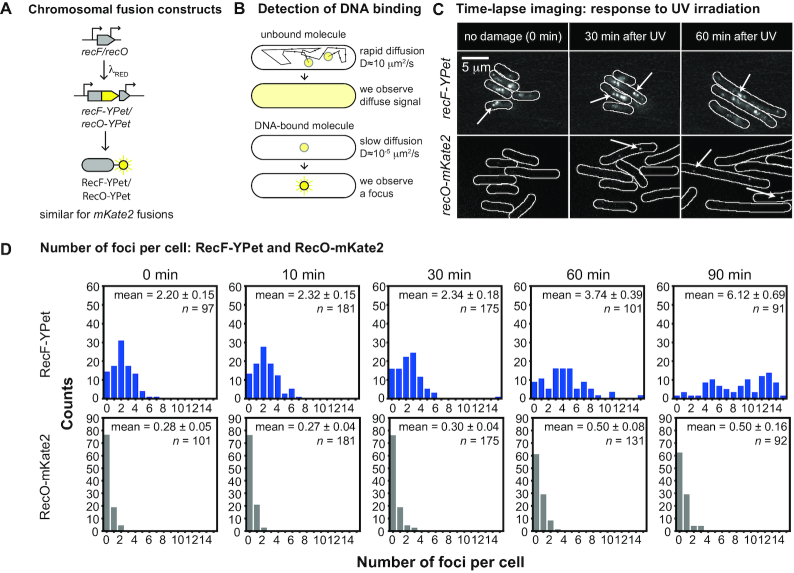
Construction and single-molecule imaging of RecF and RecO fusion constructs. (**A**) Construction of EAW670 (*recF-YPet*) and EAW814 (*recO-YPet*) as well as EAW779 (*recF-mKate2*) and EAW672 (*recO-mKate2*). The *recF* or *recO* gene of *E. coli* K12 MG1655 was modified using λ_RED_ recombineering so that RecF or RecO is expressed as a fusion with a fluorescent protein YPet or mKate2. (**B**) Detection of DNA-bound molecules in single-molecule fluorescence images. Molecules of fusion proteins that are not bound to DNA will diffuse quickly (*D* ≈ 10 μm^2^/s for a typical cytosolic protein) and thus signals from individual molecules will blur over the entire cell in our images (34- or 100-ms exposures). Molecules of fusion proteins that are bound to DNA; however, experience greatly reduced motion and thus appear as punctate foci. Because of this diffusional contrast, it is possible to detect individual molecules of RecF and RecO fusion proteins when bound to DNA. (**C**) Time-lapse imaging of RecF-YPet and RecO-mKate2 in response to UV irradiation. Cells were UV irradiated in a flow cell directly after *t* = 0 min. Images were taken from time-lapse experiments before UV irradiation (0 min) and after UV irradiation (30 and 60 min time-points); scale bar: 5 μm. (**D**) Histograms showing the number of RecF-YPet and RecO-mKate2 foci per cell in response to UV irradiation. Bright-field images were used to determine the position of cells within different fields of view. The numbers of foci per cell were counted for each cell and plotted in a histogram. We plotted these histograms for the time-point before UV irradiation (0 min) and several time-points following UV irradiation (10, 30, 60 and 90 min). The mean over the number of foci per cell is depicted in each histogram for each time-point. The number of cells that went into each histogram is indicated as *n*.

To characterize the spatiotemporal behaviour of the RecF and RecO proteins in live *E. coli* cells, we constructed functional fluorescent protein fusions of the RecF and RecO proteins to the yellow fluorescent protein (YPet) and the red fluorescent protein (mKate2) (Figure 2A, ‘Materials and methods’ section and Table 1). The activity of the RecO and RecF fusion proteins, as well as the DnaX-YPet fusion used in this work, was validated *in vivo* in several ways (Supplementary Figure S1). Briefly, all constructs used in the present study harbour similar growth, fitness, UV sensitivity and SOS induction level compared to the WT. A number of RecR fusion proteins were also constructed (Table 1). However, the fusions caused a complete loss of RecR function upon UV exposure (Supplementary Figure S1), and further work on them was not pursued.

**Table 1.** Strains used in this study

Strain	Relevant genotype	Parent strain	Source/technique
MG1655	*recF+ recO+ dnaX+*	-	(55)
EAW629	Δ*recF::kan*	MG1655	Lambda Red recombination
EAW114	Δ*recO::kan*	MG1655	Lambda Red recombination
EAW669	Δ*recR::kan*	MG1655	Lambda Red recombination
EAW20	Δ*recA::kan*	MG1655	Lambda Red recombination
EAW788	*recF(K36R)::kan*	EAW629	Lambda Red recombination
HH020	Δ*recA::kan*	MG1655	(50)
EAW670	*recF-YPet::kan*	EAW629	Lambda Red recombination
EAW779	*recF-mKate2::kan*	EAW629	Lambda Red recombination
EAW814	*recO-YPet::kan*	EAW114	Lambda Red recombination
EAW672	*recO-mKate2::kan*	EAW114	Lambda Red recombination
EAW676	*recF-YPet::FRT recO-mKate2::kan*	EAW672	Transduction of EAW672 with P1 grown on EAW670
EAW824	*recF-YPet::FRT* Δ*recO::kan*	EAW114	Transduction of EAW114 with P1 grown on EAW670
SSH068	*recF-YPet::FRT* Δ*recR::kan*	EAW670	Transduction of EAW672 with P1 grown on EAW669
SSH070	*recF-YPet::kan* Δ*recA::kan*	EAW670	Transduction of EAW672 with P1 grown on HH020
EAW822	*recO-mKate2::FRT* Δ*recF::kan*	EAW629	Transduction of EAW629 with P1 grown on EAW672
EAW697	*recO-mKate2::FRT* Δ*recR::kan*	EAW672	Transduction of EAW672 with P1 grown on EAW669
SSH067	*recO-mKate2::FRT* Δ*recA::kan*	EAW672	Transduction of EAW672 with P1 grown on HH020
JJC5945	*dnaX-YPet::kan*	MG1655	from Bénédicte Michel
CJH0015	*recF-mKate2::FRT dnaX-YPet::kan*	EAW672	Transduction of EAW672 with P1 grown on JJC5945
EAW762	*recO-mKate2::FRT dnaX-YPet::kan*	EAW672	Transduction of EAW672 with P1 grown on JJC5945
EAW826	*recF-mKate2::FRT dnaX-YPet::FRT* Δ*recO::kan*	CJH0015	Transduction of CJH0015 with P1 grown on EAW669
EAW828	*recO-mKate2::FRT dnaX-YPet::FRT* Δ*recF::kan*	EAW762	Transduction of EAW672 with P1 grown on EAW629
EAW673	*recR-mKate2::kan (Short Linker, 11 a.a.)*	EAW669	Lambda Red recombination
EAW897	*recR-mKate2::kan (Long Linker,20 a.a.)*	EAW669	Lambda Red recombination
EAW898	*recR-YPet::kan (Long Linker,20 a.a.)*	EAW669	Lambda Red recombination
EAW642	*dnaX-mKate2::kan*	MG1655	Lambda Red recombination
EAW214	Δ*araBAD*	MG1655	(51)
CJH0004	*dnaX-YPet::FRT* Δ*araBAD::kan*	JJC5945	Transduction of JJC5945 with P1 grown on EAW214
CJH0014	*recF-mKate2::FRT* Δ*araBAD::kan*	EAW779	Transduction of EAW779 with P1 grown on EAW214
CJH0010	*recF-YPet::FRT* Δ*araBAD::kan*	EAW670	Transduction of EAW770 with P1 grown on EAW214
UB2	*recO-mKate2::FRT* Δ*araBAD::kan*	EAW672	Transduction of EAW672 with P1 grown on EAW214
CJH0072	*recO-YPet::FRT* Δ*araBAD::kan*	EAW814	Transduction of EAW814 with P1 grown on EAW214
EAW1116	*recF-YPet::FRT recO-mKate2::FRT* Δ*araBAD::kan*	EAW676	Transduction of EAW676 with P1 grown on EAW214
WX31	*dnaB8*(Ts)*::kan*	AB1157	(52)
SSH114	*recF-mKate2::FRT dnaX-YPet::FRT dnaB8*(Ts)*::kan*	CJH0015	Transduction of CJH0015 with P1 grown on WX31
SSH115	*recO-mKate2::FRT dnaX-YPet::FRT dnaB8*(Ts)*::kan*	EAW762	Transduction of EAW762 with P1 grown on WX31
HG012	*Linker-mKate2* (plasmid)	MG1655	Transformation of MG1655 with pBAD-Linker-mKate2 (54)
HG013	*Linker-YPet* (plasmid)	MG1655	Transformation of MG1655 with pBAD-Linker-YPet (54)
HG362	*dnaB8*(Ts)*::kan*	MG1655	Transduction of MG1655 with P1 grown on WX31

The functional fusion constructs of the RecF and RecO proteins allowed us to generate a series of two colour strains to examine RecF and RecO within the same cell, or to examine either of these proteins in concert with the replisome. We also constructed a series of strains in which single deletions of *recO, recF, recR* or *recA*, as appropriate, were transduced into the strains encoding various fusion proteins and combinations of fusion proteins (Table 1). This was done to allow examination of the effects of such deletions on fusion protein behaviour and colocalization.

To investigate the spatiotemporal regulation of RecF and RecO proteins, we imaged single-colour strains (encoding *recF-mKate2, recF-YPet, recO-mKate2* or *recO-YPet*) in home-built flow-cells under continuous flow of oxygenated media throughout the experiment at 37°C using a custom-built single-molecule fluorescence microscope (62). Cells were irradiated with a pulse of UV light (10 J/m^2^) immediately after *t* = 0 min and imaged for 3 h after UV irradiation. In these experiments, we set out to measure three properties: (i) stoichiometry; (ii) binding lifetime and (iii) intracellular localization. We used two different single-molecule imaging modes to extract these measurements. First, burst acquisitions (movies of 300 × 34 ms, continuous excitation) enabled us to extract information on binding lifetimes, and perform photobleaching experiments used to measure stoichiometry. To measure changes in intracellular localization, we performed time-lapse imaging by collecting a snapshot of the cells every 10 min for 3 h after UV-irradiation. We also recorded a bright-field image at each time-point. All fluorescence images were recorded with single-molecule sensitivity, allowing us to observe RecF and RecO fusions binding to DNA (Figure 2B).

**Figure 3.**
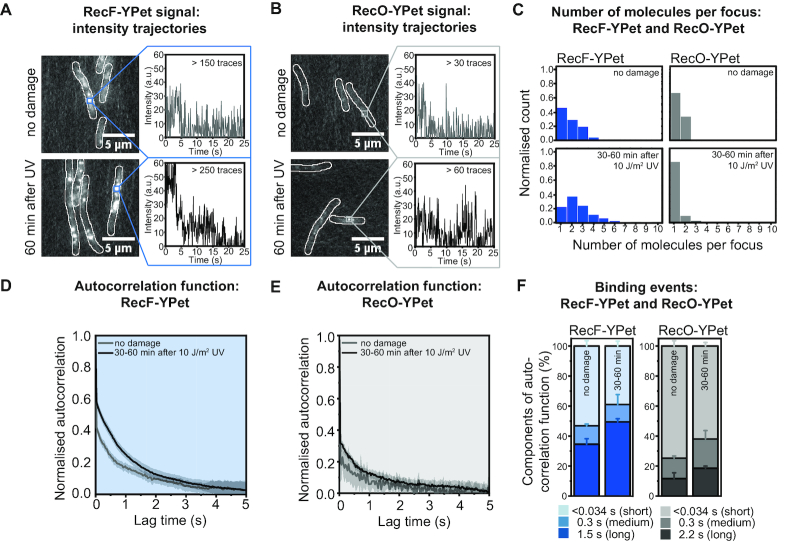
Binding behaviour of RecF-YPet and RecO-YPet to chromosomal DNA. (**A**) Average projection over time of RecF-YPet signal and representative time traces for RecF-YPet binding to DNA (continuous illumination with 34-ms exposure times over 300 frames). Average projections stem from burst acquisition movies before UV exposure and 60 min after UV exposure. The projection was made over 10 × 34 ms; scale bar: 5 μm. (**B**) Average projection over time of RecO-YPet signal and representative time traces for RecO-YPet binding to DNA (continuous illumination with 34-ms exposure times over 300 frames). Average projections stem from burst acquisition movies before UV exposure and 60 min after UV exposure. The projection was made over 10 × 34 ms; scale bar: 5 μm. (**C**) Histogram showing the number of RecF-YPet and RecO-YPet molecules per focus before UV exposure and 30–60 min after UV exposure. For the number of RecF-YPet molecules per focus before UV irradiation, 161 trajectories were sampled. For the number of RecF-YPet molecules per focus upon UV irradiation, 285 trajectories were sampled. To determine the number of RecO-YPet molecules per focus, 32 trajectories were sampled before UV exposure and 61 trajectories after UV exposure. For further explanation, see Supplementary Figure S3. (**D**) Autocorrelation function obtained for RecF-YPet binding events before and after UV exposure. For further explanation, see Supplementary Figure S4. (**E**) Autocorrelation function obtained for RecO-YPet binding events before and after UV exposure. For further explanation see Supplementary Figure S4. (**F**) Components of the autocorrelation for RecF-YPet and RecO-YPet binding to DNA. Components of the autocorrelation function for RecF-YPet before and after UV exposure are long (1.5 s), medium (0.3 s) and short (<0.034 s). For RecO-YPet, components are split in long (2.2 s), medium (0.3 s) and short (<0.034 s). Error bars for long and medium components are derived from the exponential fit (Supplementary Figure S4), error bars for short events stem from the standard error of the mean at lag time 0 s.

When recording time-lapse data in the absence of DNA damage, we observed punctate foci of RecF-YPet, consistent with RecF-YPet molecules binding to DNA (Figure 2C). On average, cells contained 2.2 ± 0.2 RecF-YPet foci (Figure 2D). Similarly, RecF-mKate2 cells contain 1.7 ± 0.1 foci per cell (Supplementary Figure S2). We then investigated the binding behaviour of RecF-YPet more closely. Using burst acquisition measurements, we observed RecF-YPet molecules binding to DNA while others were freely diffusing (Figure 3A). We extracted fluorescence intensity trajectories from binding events that lasted several hundreds of milliseconds (>150 trajectories) (Supplementary Figure S3). Trajectories featured prominent bleaching steps due to the continuous exposure to excitation light, each step representing a single YPet molecule that has bleached. The distribution of intensity steps was used to determine the intensity equivalent to one RecF-YPet molecule (Supplementary Figure S3). Knowing the intensity of a single RecF-YPet molecule, we determined that RecF foci predominantly contain one molecule per focus in undamaged cells (Figure 3C). Brighter foci could correspond to oligomers of RecF (i.e. dimers, trimers…) or multiple RecF monomers producing overlapping foci.

Intensity traces were further used to investigate the time scale on which RecF-YPet molecules are bound to DNA (Supplementary Figure S4). To investigate the time scale of binding events, we utilized autocorrelation analysis, a method that identifies time-dependent fluctuations in signal which are also dependent on binding and dissociation of molecules. When applying the autocorrelation function to a RecF-YPet trajectory, the correlation of this trajectory with its time delayed copy is generated for various lag times. With zero lag time, the normalized correlation of a trajectory with itself is one. After zero lag time, RecF-YPet molecules that are statically bound would give autocorrelation values between zero and one depending on the signal-to-noise. However, RecF-YPet molecules that are transiently associated show zero autocorrelation. Autocorrelation analysis can thus be used to identify major components of binding events. We generated an average over all autocorrelation functions for each condition (before and after UV irradiation) which was then used to extract information on the overall binding behaviour (Figure 3D and Supplementary Figure S4). The averaged autocorrelation function contained three major components reflecting multiple time-dependent processes present in the signal. The first was a fast decorrelation occurring on the time scale of the integration time (an individual camera frame exposure, one frame of the burst acquisition) attributable to noise as well as, transient binding events that occur within the time resolution of imaging. This fast decorrelation component is hereafter referred to as the short component (*τ*_s_ < 0.034 s). Fitting the averaged autocorrelation curve starting from lag time 0.034 s (after the initial fast decorrelation) with single and double exponential-decay functions indicated that there were two major component timescales present in the remainder of the autocorrelation curve (Supplementary Figure S4 shows two-exponential fit). In both undamaged as well as damaged cells (30–60 min after UV), the fluorescence signal decayed according to two timescales: medium corresponding to 0.3 s (*τ*_m_) and long corresponding to 1.5 s (*τ*_l_) reflecting longer lived binding events. The amplitudes of these decay functions in the autocorrelation function for RecF-YPet are 53% short (*a*_s_), 12% medium (*a*_m_) and 35% long (*a*_l_).

Our experiments also enabled us to further determine the cellular concentration of RecF-YPet. Knowing the intensity of a single YPet molecule from our trajectories, we calculated that there were 18.1 ± 0.7 molecules of RecF-YPet per cell (standard deviation STD = 5.5; *n* = 71 cells) (Supplementary Figure S5), equivalent to a RecF-YPet concentration of 5.4 ± 0.2 nM (‘Materials and methods’ section). From the above measurements (18 molecules per cell, two foci, one molecule per focus), we concluded that ∼11% of RecF-YPet molecules were bound to DNA at any given moment in the absence of DNA damage.

We undertook the same measurements for EAW814 (*recO-YPet)* and EAW672 (*recO-mKate2*). RecO foci were much less common than RecF foci. Using time-lapse measurements (100-ms exposure), we determined that only three in ten cells have a RecO-mKate2 focus (Figure 2D). Consistent with these measurements, cells expressing a RecO-YPet fusion (EAW814) contain on average 0.4 ± 0.04 foci per cell (Supplementary Figure S2). Burst acquisition measurements showed that most RecO-YPet molecules are diffusive and a RecO-YPet molecule binds to DNA only occasionally (Figure 3B). These RecO-YPet foci contain one molecule per focus (Figure 3C and Supplementary Figure S3). RecO-YPet binding events were then analysed using autocorrelation analysis. The components of the autocorrelation function were 75% short (*a*_s_, *τ*_s_ < 0.034 s), 13% medium (*a*_m_, *τ*_m_ = 0.3 s) and 12% long (*a*_l_, *τ*_l_ = 2.2 s) (Figure 3E and F; Supplementary Figure S4). We further determined that cells have 12.2 ± 0.6 RecO-YPet molecules per cell (STD = 5.9; *n* = 98 cells), corresponding to a RecO-YPet concentration of 3.7 ± 0.2 nM (Supplementary Figure S5). With only 0.3 foci per cell and 12 RecO-YPet molecules per cell, only ∼2% of RecO molecules are DNA bound at any given moment in the absence of any cellular stress.

Next, we investigated the behaviour of RecF and RecO fusions in cells damaged with 10 J m^−2^ of UV light. Using time-lapse measurements, we observed that cells filament after acquiring UV induced DNA damage, beginning ∼30 min after UV irradiation (Supplementary Figure S6A). We further determined the mean pixel intensities within cell boundaries (mean cell intensity) to identify possible changes in the concentration of RecF-YPet upon DNA damage induction. We found that the mean cell intensity is constant during the experiment, indicating that the concentration of RecF-YPet remains constant throughout the experiment (Supplementary Figure S6B). As cells grow into filaments, more RecF-YPet molecules bind to DNA (Figure 2C and D), for instance, cells have approximately six RecF-YPet foci per cell at 90 min. We calculated the focus density (foci per cell area) using the time-lapse data. Even though the number of binding sites increases for RecF-YPet, the focus density is constant before and after UV irradiation as the number of binding sites increases proportionally with the increase in cell length (Supplementary Figure S6C). In contrast to untreated cells, however, RecF-YPet foci contain approximately two molecules per focus starting 30 min after UV irradiation (Figure 3A and C). This suggests that RecF forms a dimer, a molecular form previously characterized (46,66–68), in response to UV irradiation. From autocorrelation analysis, we identified that more RecF molecules seem to bind slightly longer to DNA 30–60 min after UV irradiation. The components of the autocorrelation function are 38% short (*a*_s_, *τ*_s_ < 0.034s), 12% (*a*_m_, *τ*_m_ = 0.3 s) medium and 50% long (*a*_l_, *τ*_l_ = 1.5s) (Figure 3D and F). There are (at least) two possible explanations for the difference in RecF binding behaviour between untreated and UV-irradiated cells. More RecF molecules may bind on the longer timescale to DNA after UV irradiation. Alternatively, the formation of RecF-YPet dimers observed after UV is associated with an increase in focus intensity. This increase in intensity causes an increase in the signal-to-noise ratio for RecF foci which then decreases the rapid (short) component of the autocorrelation curve. Analysis of simulated data suggests that the second case is likely (Supplementary Figure S7; ‘Materials and methods’ section). With RecF forming a dimer and cells exhibiting a constant focus density and mean cell intensity, ∼22% of RecF-YPet molecules are DNA bound after damage induction. This is a 2-fold increase compared to untreated cells and is driven primarily by dimerization of RecF rather than an increase in the density of binding sites on the DNA.

As observed for cells expressing RecF fusion proteins, cells carrying RecO fusion constructs grow into filaments upon UV irradiation (Figure 2C and Supplementary Figure S6A). The mean cell intensity derived from the fusion proteins stays constant over time (Supplementary Figure S6B) suggesting no change in the cellular concentration of RecO. As cells grow into filaments upon UV irradiation, cells contain more RecO foci (Figure 2C and D; Supplementary Figure S2) while the focus density remains constant over time (Supplementary Figure S6C). In contrast to RecF-YPet foci, RecO-YPet foci consist of only one molecule per focus and thus are monomeric before and after UV damage (Figure 3B and C). UV irradiation results in a small increase in the number of long-lived RecO foci; the components of the autocorrelation function were 62% short (*a*_s_, *τ*_s_ < 0.034 s), 20% (*a*_m_, *τ*_m_ = 0.3 s) medium and 18% long (*a*_l_, *τ*_l_ = 2.2s) (Figure 3E and F). Since the focus density and mean cell brightness are constant and RecO foci are still monomeric after UV irradiation, ∼2% of RecO-YPet molecules are DNA bound both before and after DNA damage induction.

### RecF and RecO exhibit different spatiotemporal behaviour

**Figure 4.**
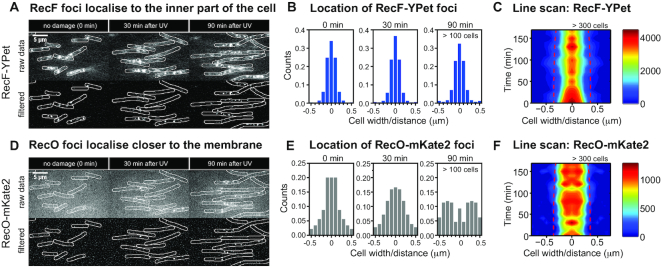
Spatiotemporal behaviour of RecF-YPet and RecO-mKate2 following UV treatment. (**A**) Time-lapse imaging of RecF-YPet in response to UV irradiation. Cells were UV irradiated in a flow cell directly after *t* = 0 min. Images were taken from time-lapse experiments before UV irradiation (0 min) and after UV irradiation (30 and 90 min time-points); scale bar: 5 μm. (**B**) Histogram showing the localization of RecF foci along the short axis of the cell. Histograms are derived from ∼100 cells at each time-point (for exact numbers, see Figure 2). The centre spline of the cell (a line drawn down the long axis) is at 0 μm, the cell membrane is at 0.5 and −0.5 μm. (**C**) 2D contour plot showing the spatiotemporal behaviour of RecF-YPet following the SOS response. The cell width is given in micrometres, the mid-cell position is at 0 μm and the dashed red line indicates the signal of a membrane binding protein, LacY. High focus abundance and other high-spatial frequency features are shown by red coloured areas in the localization map; low focus abundance is illustrated by blue coloured areas. (**D**) Time-lapse imaging of RecO-mKate2 in response to UV irradiation. For further description see panel (A); scale bar: 5 μm. (**E**) Histogram showing the localization of RecO foci along the short axis of the cell. For further description, see panel (B). (**F**) 2D contour plot showing the spatiotemporal behaviour of RecO-mKate2 following the SOS response. For further description, see panel (C).

We further defined the spatiotemporal behaviour of RecF and RecO in response to UV damage. This was achieved through two-colour time-lapse imaging of EAW676 (*recF-YPet recO-mKate2*). Cells were irradiated with a UV dose of 10 J/m^2^ directly after *t* = 0 min and imaged for a period of 3 h after UV irradiation. Images were recorded once every 10 min (Figure 4A and D).

When analysing the spatial localization of RecF in response to DNA damage, we examined whether foci localize within the inner part of the cell or closer to the membrane (focus position along the cellular width). We plotted histograms of the RecF foci position with respect to the short axis of the cell (i.e. width) prior to damage induction, as well as 30 and 90 min after UV irradiation (Figure 4B). The centre spline of the cell (a line drawn down the long axis) is at 0 μm, the cell membrane is at 0.5 and −0.5 μm. We found that RecF foci localize predominantly within the inner part of the cell before and after UV irradiation. The vast majority of the RecF foci were located within 0.2 μm of the cell centre. To further characterize the spatiotemporal localization of RecF throughout the experiment, we used a tool that yields information on the distributions of sparse fluorescence signals by averaging signals across cross-sections of many cells (69). The resulting data are referred to as line scans and represent the average fluorescence intensity across the short axis of the cell. Prior to analysis, we enhanced the focus intensity and reduced the background signal using digital filters (61). High intensity areas within cells thus represent foci and other high-spatial frequency features. Using our time-lapse data, this tool plots a 2D contour plot showing the spatiotemporal behaviour of RecF-YPet following the SOS response (Figure 4C). The cell width is given in micrometres, whereas, the mid-cell position is at 0 μm and the dashed red line indicates the signal of a membrane binding protein, LacY (61). High focus abundance is shown by red coloured areas in the localization map; low focus abundance is illustrated by blue coloured areas. We found that RecF foci are localized to the inner part of the cell before and after damage induction. This localization behaviour has previously been found for replisome markers following UV irradiation (69).

We also investigated the spatiotemporal behaviour of RecO. In comparison with RecF, RecO produces a broader distribution around 0 μm prior to UV irradiation (Figure 4E). After UV irradiation, the distribution broadened further. At 30 min, more foci were localized closer to the membrane. At 90 min, most RecO foci were localized in proximity to the membrane. Two broader peaks appeared at the −0.3 and 0.3 μm position, with relatively few foci found at the 0 μm position. When plotting the 2D contour plot showing the spatiotemporal behaviour of RecO-mKate2, we observed that the distribution broadened 30–50 min after damage induction, corresponding closely to the time when cells begin to grow into filaments (Figure 4F). This reveals that RecO usually binds at positions closer to the membrane following the SOS response, likely excluded from the nucleoid.

### RecF and RecO foci localize differently with respect to replisome markers

**Figure 5.**
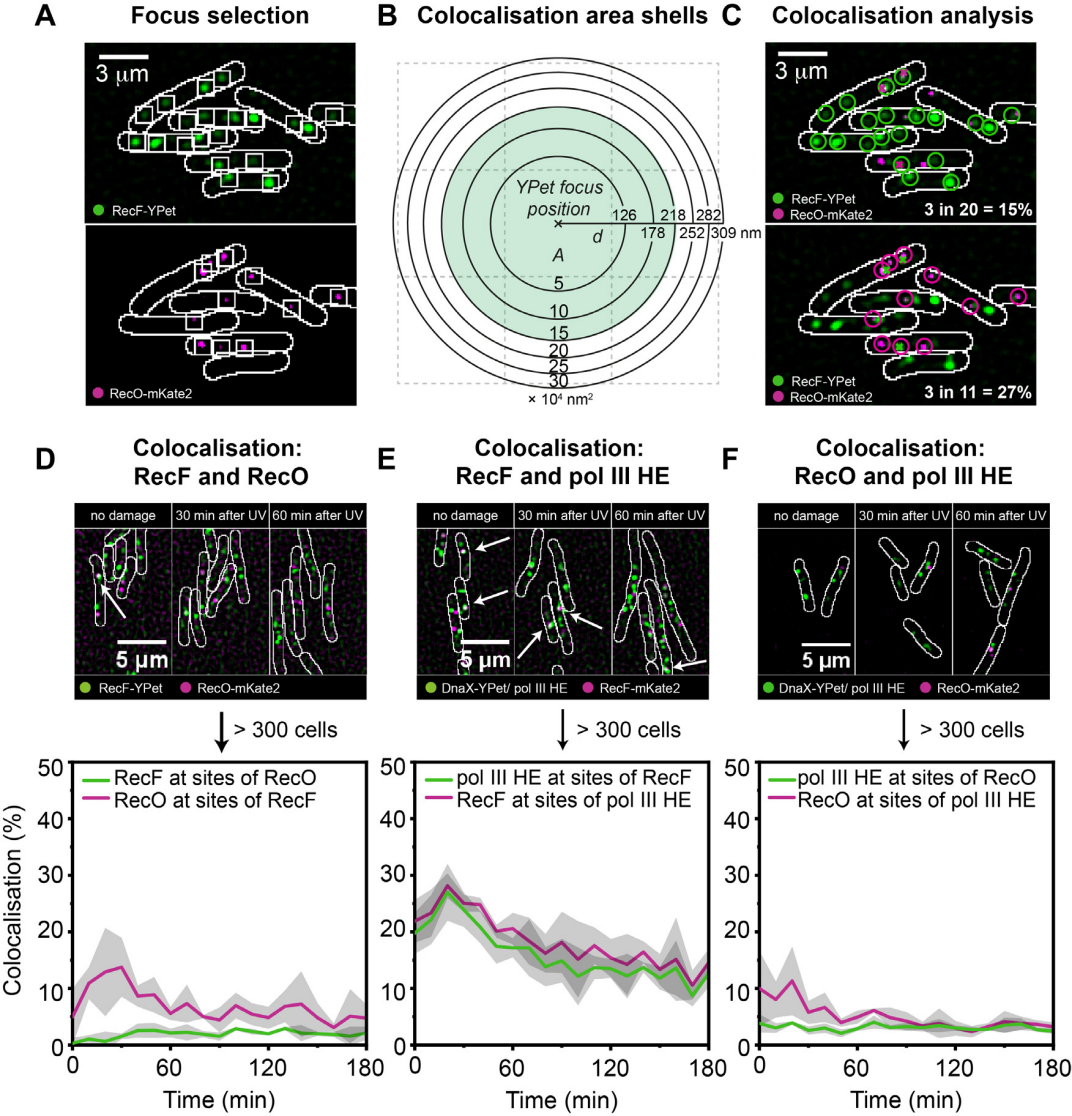
Colocalization measurements of RecF/RecO, RecF/replisomes and RecO/replisomes. (**A**) Exemplary selection of RecF-YPet and RecO-mKate2 foci. Selection boxes indicate selected foci for recF-YPet and RecO-mKate2; scale bar: 3 μm. (**B**) Diagram of area shells used for colocalization analysis. As colocalization is a radial measurement, histograms of colocalization distances are constructed using bins of linearly increasing area rather than distance. A colocalization radius of 218 nm was used for all measurements since two replisome components colocalize within this colocalization radius. (**C**) Montage of two-colour images shown in (A). RecF-YPet foci appear in green and RecO-mKate2 foci appear in magenta. Upper panel: Colocalization percentages for RecF-YPet with RecO-mKate2 are determined from selected foci in the RecF-YPet channel (green circles) that colocalize to the same position with RecO-mKate2 foci from the other channel (magenta crosses). Lower panel: The opposite is shown to determine colocalization percentages of RecO-mKate2 (magenta circles) with RecF-YPet (green crosses); scale bar: 3 μm. (**D**) Colocalization measurements of RecF-YPet with RecO-mKate2 in response to 10 J/m^2^ UV. Merged images of RecF-YPet (green signal) and RecO-mkate2 (magenta signal) are shown before UV irradiation and after UV irradiation (30 and 60 min). Colocalization was measured over >300 cells. The percentage of RecF-YPet foci that contain a RecO-mKate2 focus is plotted as a green line plot over 180 min at intervals of 10 min. Similarly, the colocalization of RecO-mkate2 with RecF-YPet is plotted as a magenta line plot; scale bar: 5 μm. (**E**) Colocalization measurements of RecF-mKate2 with DnaX-YPet (replisomes) in response to 10 J/m^2^ UV. Merged images of RecF-mKate2 (magenta signal) and DnaX-YPet (green signal) are shown before UV irradiation and after UV irradiation (30 and 60 min). The percentage of RecF-mKate2 foci that contain a DnaX-YPet focus is plotted in green, the percentage of DnaX-YPet that colocalize with RecF-mKate2 is depicted with a magenta line plot (*n* > 300 cells); scale bar: 5 μm. (**F**) Colocalization measurements of RecO-mKate2 with DnaX-YPet (replisomes) in response to 10 J/m^2^ UV. Merged images of RecO-mkate2 (magenta signal) and DnaX-YPet (green signal) are shown before UV irradiation and after UV irradiation (30 and 60 min). Colocalization of RecF-mKate2 with DnaX-YPet is illustrated by a green line plot; colocalization of DnaX-YPet with RecO-mKate2 is presented by a magenta line plot (*n* > 300 cells); scale bar: 5 μm.

Due to strong differences in the spatiotemporal behaviour of RecF and RecO, we wished to determine if there was any indication that RecF and RecO formed a complex *in vivo*, as indicated by a sharing of chromosomal binding sites. We determined the percentage of RecF foci that colocalized with RecO foci and the percentage of RecO foci that colocalized with RecF foci following the SOS response. For colocalization analysis, we selected foci for each of the proteins that are each labelled with a different fluorescent protein (i.e. RecF-YPet and RecO-mKate2, Figure 5A). We defined two foci (i.e. a RecF and a RecO focus) as colocalized if their centroid positions were within 218 nm of each other (Figure 5B and C). This distance corresponds to the maximum colocalization distance observed between two replisome probes, which are expected to be highly colocalized (62). To conduct colocalization analysis of RecF and RecO, we used the two-colour time-lapse data of EAW676 (*recF-YPet recO-mKate2*) inducing UV damage directly after *t* = 0 min.

In undamaged cells, only 0.5% of RecF foci also contained a RecO focus (chance colocalization <1%, ‘Materials and methods’ section) while 5% of RecO foci had a coincident RecF focus (chance colocalization ∼4%, ‘Materials and methods’ section) (Figure 5D). Note that the calculated frequency of chance colocalization takes into account the fact that many cells do not have RecO foci, but most have RecF foci. After exposure to 10 J/m^2^ UV, the percentage of RecF foci that are coincident with a RecO focus slightly increased to ∼2% at 40 min after damage induction. The colocalization of RecO with RecF increased to 12% at 30–40 min followed by a gradual drop in colocalization to 5% at 50 min. Following the SOS response, the colocalization of RecF with RecO was just above the level calculated for chance colocalization, whereas colocalization of RecO with RecF is slightly above chance in the 10–50 min time interval. Our data clearly suggest that RecF and RecO have predominantly distinct binding sites both before and after exposure to UV, and provide no evidence for a RecFOR complex.

We further examined if RecF and RecO localized to the replisome. We performed two-colour time-lapse experiments and colocalization analysis by imaging CJH0015 (*recF-mKate2 dnaX-YPet*) and EAW762 (*recO-mKate2 dnaX-YPet*) as described above (UV dose: 10 J/m^2^ just after *t* = 0 min; images were taken every 10 min for 3 h after UV, experiments were conducted at 37°C).

We observed that the colocalization of RecF with the replisome marker DnaX-YPet was quite significant, both before and after UV irradiation (Figure 5E). Before damage induction, colocalization of RecF with the replisome marker was 22% (chance colocalization ∼4%, ‘Materials and methods’ section). Similarly, 20% of replisome foci contained a RecF focus (chance colocalization ∼4%, ‘Materials and methods’ section). After UV irradiation, the percentage of RecF foci that contained a replisome focus peaked at 30% at 30 min. This peak was followed by a gradual decline in colocalization, and at 120 min after UV irradiation only 15% of RecF foci overlapped with a replisome focus. The colocalization of replisomes with RecF followed the same trend upon UV irradiation. At 30 min, 27% of replisome foci contained a RecF focus. At 120 min, 13% of replisome foci had a RecF focus. RecF appeared to be recruited to replisomes directly after UV exposure. In general, RecF displayed relatively high colocalization with replisome markers, suggesting that RecF function often involves action at, or near, replisomes.

In contrast to colocalization measurements between RecF and replisomes, RecO rarely bound at sites of replisomes (Figure 5F). In undamaged cells, 10% of RecO foci colocalized with replisomes (chance colocalization ∼4%, ‘Materials and methods’ section); 4% of replisome foci contained a RecO focus (chance colocalization <1%, ‘Materials and methods’ section). Colocalization between RecO and replisomes progressively decreased after UV irradiation; only 3% of RecO foci contained a replisome focus at 120 min, a level below that expected by chance. Thus, the vast majority of RecO foci (97%) are spatially distinct from replisomes. The percentage of replisomes containing a RecO molecule remained at 3–4% throughout the experiment, constantly just above the level calculated for chance colocalization. This suggests that RecO binding sites, and sites of action, rarely correspond with replisomes in cells.

### RecF and RecO function independently of each other

To investigate if RecF and RecO act independently, we first determined the number of RecF foci in Δ*recO* cells and the number of RecO foci in Δ*recF* cells at 37°C. A slower cell filamentation rate is associated with a slower increase in the number of foci. In these experiments, we used cell filamentation as a proxy for SOS induction.

**Figure 6.**
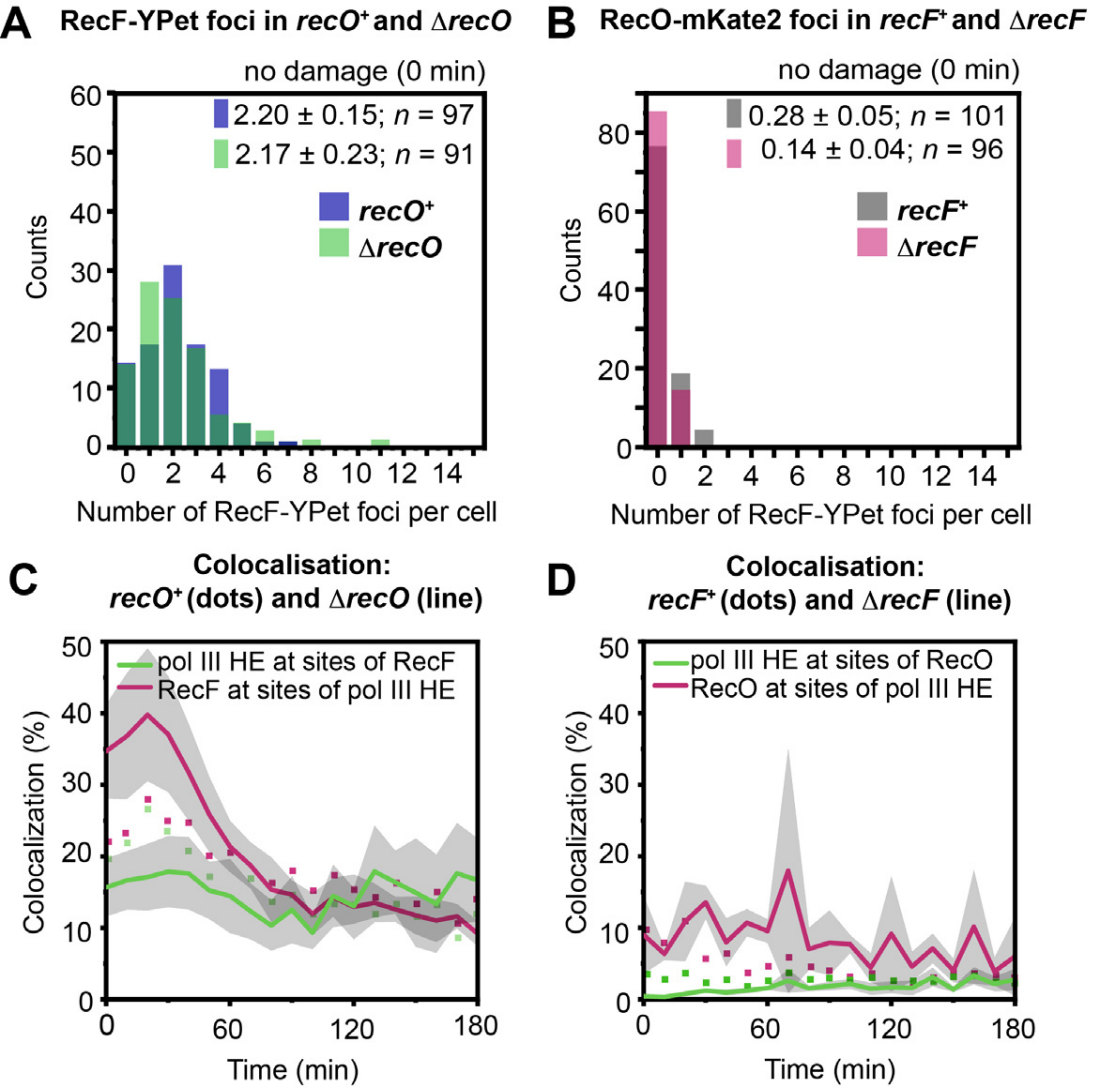
Colocalization measurements of RecF with replisomes in Δ*recO* and RecO with replisomes in Δ*recF*. (**A**) Histograms showing the number of RecF-YPet foci per cell in Δ*recO* (green) and *recO^+^* (blue) under normal growth conditions. Bright-field images were used to determine the position of cells within different fields of view. The numbers of foci per cell were counted for each cell and plotted in a histogram. The mean over the number of foci per cell is given in each histogram. The number of cells included in each histogram is also indicated as *n*. (**B**) Histograms showing the number of RecO-mKate2 foci per cell in Δ*recF* (pink) and *recF^+^* (grey) under normal growth conditions. Bright-field images were used to determine the position of cells within different fields of view. The numbers of foci per cell were counted for each cell and plotted in a histogram. The mean over the number of foci per cell is given in each histogram. The number of cells included in each histogram is also indicated as *n*. (**C**) Colocalization measurements of RecF-mKate2 with DnaX-YPet (replisomes) in Δ*recO* following 10 J/m^2^ UV. The percentage of RecF-mKate2 foci that contain a DnaX-YPet focus is plotted in green, the percentage of DnaX-YPet that colocalizes with RecF-mKate2 is depicted with a magenta line plot (*n* > 100 cells). The colocalization of RecF-mKate2 with DnaX-YPet in *recO^+^* (magenta scatter plot), and the colocalization of DnaX-YPet with RecF-mKate2 in *recO^+^* (green scatter plot) is also plotted for each time-point as in Figure 5E. (**D**) Colocalization measurements of RecO-mKate2 with DnaX-YPet (replisomes) in Δ*recF* following 10 J/m^2^ UV. The percentage of RecO-mKate2 foci that contain a DnaX-YPet focus is plotted in green, the percentage of DnaX-YPet that colocalizes with RecO-mKate2 is depicted with a magenta line plot (*n* > 100 cells). The colocalization of RecO-mKate2 with DnaX-YPet in *recF^+^* (magenta scatter plot) and the colocalization of DnaX-YPet with RecO-mKate2 in *recF^+^* (green scatter plot) are also plotted for each time-point as in Figure 5F.

In the absence of DNA damage, we found that the deletion of *recO* did not affect the number of RecF foci (Figure 6A and Supplementary Figure S8). After damage induction, cells lacking *recO* filamented slower than wild-type cells. A subset of cells within the population were static and did not grow into filaments (Supplementary Figure S10). The mixed population of slowly filamenting cells and static cells produced a broad distribution in cell length beginning about 30 min after UV irradiation. We further observed that the number of RecF-YPet foci in Δ*recO* cells increased slower than in *recO*^+^ cells (Supplementary Figure S11). This result agrees with our previous observation that the increase in cell length is associated with an increase in number of foci (i.e. the focus density is constant, Supplementary Figure S6).

The deletion of *recF* marginally lowered the number of RecO foci before UV irradiation (Supplementary Figure S9). Cells lacking *recF* also filamented slower than wild-type cells upon UV treatment. However, we did not detect a static population that does not grow into filaments as seen for cells lacking *recO* (Supplementary Figure S10). The number of RecO foci in Δ*recF* increases steadily as cells grow into filaments, remaining just slightly lower than in wild-type cells (Supplementary Figure S12).

To determine if the activity of RecF at replisomes is independent of RecO and vice versa, we conducted colocalization measurements for RecF and replisomes in the Δ*recO* background as well as RecO and replisomes in the Δ*recF* background. Time-lapse experiments (10 J/m^2^ directly after 0 min, at 37°C) and colocalization measurements were conducted as described above.

Colocalization measurements of RecF with the replisome in Δ*recO* cells returned higher extents of colocalization while retaining the trend observed for wild-type cells (Figure 6C). In the absence of damage, 35% of RecF foci were colocalized with a replisome. At 30 min, colocalization peaked at 40% followed by a slow decrease in colocalization (chance colocalization ∼4%). From 90 min, 14% of RecF foci were coincident with a replisome focus. When measuring colocalization between the replisome and RecF in Δ*recO*, 16% of replisomes had a RecF focus bound before UV irradiation, which is slightly lower than in wild-type cells (chance colocalization ∼4%). After UV irradiation, colocalization marginally increased to 18% at 30 min, followed by a slight drop to 13% at 90 min.

The deletion of *recF* only marginally changed the colocalization behaviour of RecO with replisomes. In the absence of damage, 9% of RecO foci contained a replisome focus (Figure 6D) as seen for wild-type cells. During the experiment, the percentage of RecO foci that contained a replisome stays on average at ∼8% which was just above chance (chance colocalization ∼4%). In the *recF^+^* background, the small degree of RecO-replisome colocalization present in the absence of damage dropped below chance after UV irradiation. This drop did not appear to occur in the Δ*recF* background. We then measured the colocalization of replisomes with RecO foci; 0.5% of replisomes contained a RecO focus in the absence of damage. The colocalization percentage stayed low following the SOS response. From 60 min, only 2% of replisomes contained a RecO focus (chance colocalization <1%).

Thus, independently of RecO, RecF is recruited to replisomes directly after UV irradiation while the number of RecF foci per cell slowly increases. Similarly, the number of RecO foci per cell increases upon UV irradiation independently of RecF. In *recF^+^* and Δ*recF*, RecO predominantly binds at sites that are spatially distinct from replisomes, in both untreated and UV-irradiated cells.

### RecF and RecO functions exhibit differences in RecR dependency

We further determined the number of RecF foci in Δ*recA* and Δ*recR* cells (Supplementary Figures S8 and S11) and the number RecO foci in Δ*recA* and Δ*recR* cells (Supplementary Figures S9 and S12). We also monitored cell length as a function of time, since *recO* and *recR* deletions interfere with RecA loading which presumably changes the upregulation of the SOS response and could delay filamentation (Supplementary Figure S10).

In the absence of DNA damage, cells lacking *recA* (SSH067, SSH070) exhibited normal size (Supplementary Figure S10) and numbers of RecF or RecO foci per cell were comparable to wild-type cells (Supplementary Figure S11). Upon UV irradiation, cells lacking *recA* divided once and then remained static, or stayed at the same size from the time of exposure. Most cells however remained static (>90%, Supplementary Figure S10). In addition, UV irradiation caused a reduction in RecF and RecO foci (Supplementary Figures S11 and S12). At 90 min after irradiation, cells lacking RecA contained on average 0.9 ± 0.1 RecF and 0.13 ± 0.05 RecO foci per cell, whereas wild-type cells contained 6.1 RecF and 0.5 RecO foci per cell.

When deleting *recR*, cells exhibited a filamentation behaviour more similar to cells lacking *recO* than cells lacking *recF*; they either filament slowly or stay at the same size (Supplementary Figure S10). Cells lacking *recR*, however, show differences in the number of RecF and RecO foci compared to *recR^+^* cells (Supplementary Figures S11 and S12). Before damage induction, *recR* mutants contained approximately 1.3 ± 0.1 RecF foci per cell (*recR^+^*: 2.2 ± 0.2 foci) and 0.11 ± 0.04 RecO foci per cell (*recR^+^*: 0.3 ± 0.1 foci). Upon UV irradiation, mutants of *recR* contain fewer RecF foci on average (0.9 ± 0.1 foci). Static cells lose their RecF foci, whereas filamented cells have an increased number of RecF foci which is still lower than in wild-type cells (Supplementary Figure S8). Overall, cells lacking *recR* exhibit a lower number of RecF foci which agrees with previous studies; RecR increases the binding affinity of RecF to DNA, suggesting that a RecFR complex is important for some processes *in vivo* (11,43,44). In contrast, the average number of RecO foci per cell remained low but did not change significantly with respect to *recR^+^*cells. At 90 min after UV irradiation, *recR* mutants contained 0.4 ± 0.1 RecO foci per cell, which is similar to *recR^+^*cells containing 0.5 ± 0.2 foci per cell. This suggests that RecO often binds its substrates independently of RecR. At this stage, it is not clear whether this behaviour represents a simple non-productive binding event, perhaps to SSB, an event that leads to RecA loading, or another undiscovered function.

### RecF and RecO form foci only under conditions of active DNA replication

**Figure 7.**
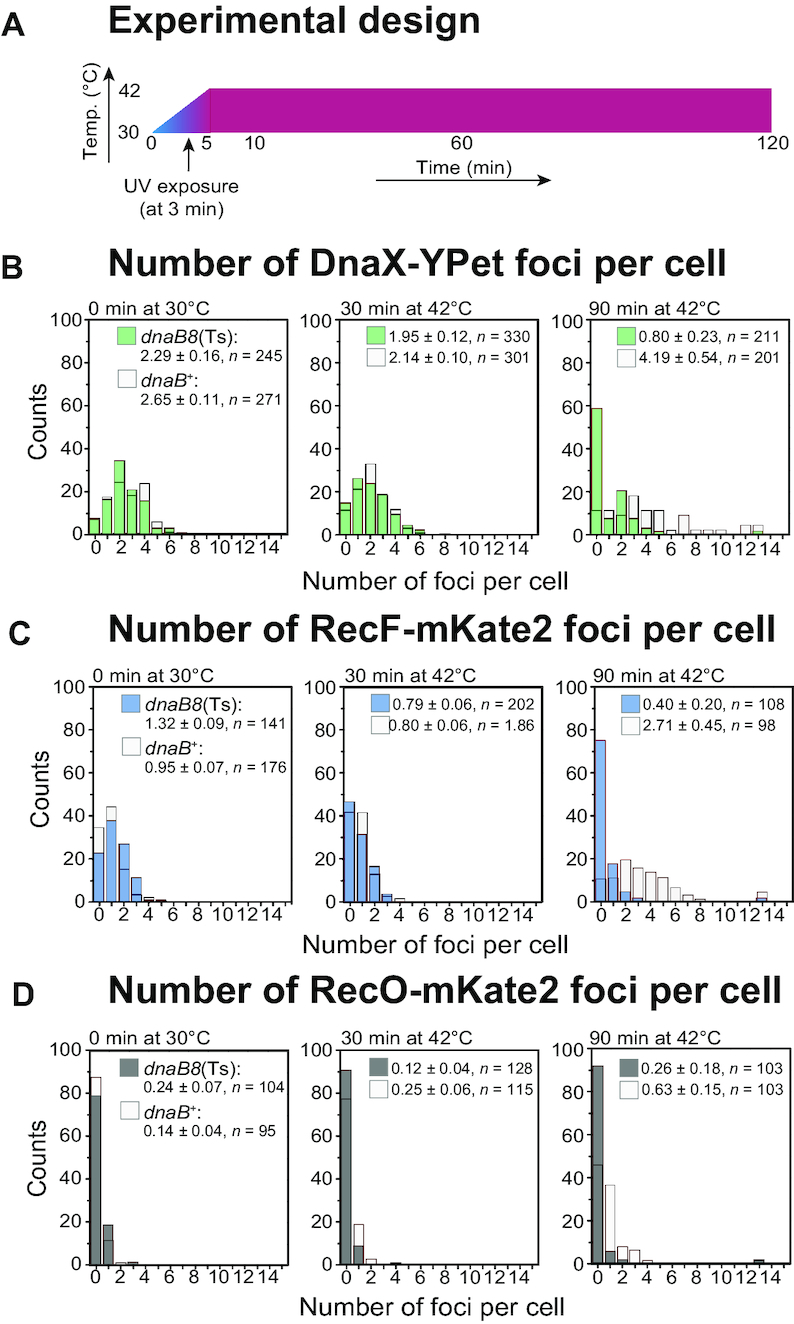
Number of DnaX-YPet, RecF-mKate2 and RecO-mKate2 foci per cell in replicating cells (*dnaB*^+^) and cells experiencing replication blocking (*dnaB8*[Ts]). (**A**) Experimental design. First image is taken at 30°C (0 min) when no UV damage is yet induced. Then, the temperature is ramped up to 42°C. UV damage is induced at 3–4 min. At 42°C, they are reached at 5 min and hold until the end of the experiment, at 120 min. (**B**) Histograms showing the number of DnaX-YPet foci per cell in *dnaB^+^* (light grey) and *dnaB8*(Ts) (green) before UV exposure, at 30°C (0 min) and after UV exposure at 42°C (30 and 90 min). Bright-field images were used to determine the position of cells within different fields of view. The numbers of foci per cell were counted for each cell and plotted in a histogram. The mean over the number of foci per cell is given in each histogram. The number of cells included in each histogram is also indicated as *n*. (**C**) Histograms showing the number of RecF-mKate2 foci per cell in *dnaB*^+^ (light grey) and *dnaB8*(Ts) (blue) before UV exposure, at 30°C (0 min) and after UV exposure at 42°C (30 and 90 min). Bright-field images were used to determine the position of cells within different fields of view. The numbers of foci per cell were counted for each cell and plotted in a histogram. The mean over the number of foci per cell is given in each histogram. The number of cells included in each histogram is also indicated as *n*. (**D**) Histograms showing the number of RecO-mKate2 foci per cell in *dnaB*^+^ (light grey) and *dnaB8*(Ts) (dark grey) before UV exposure, at 30°C (0 min) and after UV exposure at 42°C (30 and 90 min). Bright-field images were used to determine the position of cells within different fields of view. The numbers of foci per cell were counted for each cell and plotted in a histogram. The mean over the number of foci per cell is given in each histogram. The number of cells included in each histogram is also indicated as *n*.

Recombination via the RecFOR pathway is thought to be the major mechanism for the resolution of post-replicative ssDNA gaps in bacteria. We reasoned that if the majority of RecF and RecO foci observed in our experiments represent proteins engaged in post-replicative gap repair, then blocking DNA replication should reduce the number of RecF and RecO foci. To test this hypothesis, we first constructed strains that carry a temperature sensitive *dnaB* allele in place of the wild-type allele (52,53): SSH114 (*recF-mKate2 dnaX-YPet dnaB8*[Ts]) and SSH115 (*recO-mKate2 dnaX-YPet dnaB8*[Ts]). Next, we conducted two-colour time-lapse experiments in which we observed the ability of tagged RecF and RecO proteins to form foci in UV-irradiated cells following a rapid jump from the permissive to the non-permissive temperature (Figure 7; Supplementary Figures S13 and S14). To that end, we first collected data at the permissive temperature at the first time-point (*t* = 0 min, *T* = 30°C). Following this acquisition, cells were irradiated with a UV-dose of 10 J m^−2^ and imaged every 10 min for 2 h. In these experiments, the stage temperature changed from 30 to 42°C within 5 min, such that the temperature of the flow cell was 42°C for imaging time-points *t* = 10–120 min (Figure 7A). Additionally, we repeated these experiments with cells carrying the wild-type *dnaB* allele.

From the time-lapse images, we then measured the number of DnaX foci per cell as a proxy for active DNA replication forks. As expected, both wild-type and temperature sensitive cells exhibited identical number of DnaX-YPet foci (*dnaB^+^*: 2.65 ± 0.11, *dnaB8*(Ts): 2.29 ± 0.16) prior to UV irradiation at the permissive temperature (Figure 7B). Following UV damage, whereas both *dnaB8*(Ts) and *dnaB*^+^ cells exhibited classic cell filamentation that accompanies the triggering of the SOS response (Supplementary Figure S13). The number of DnaX foci per cell decreased in *dnaB8(Ts)* while the number of DnaX foci increased in *dnaB*^+^ cells (Figure 7B and Supplementary Figure S13). At 90 min after irradiation, cells contain on average 0.80 ± 0.23 DnaX foci in the *dnaB8*(Ts) background and 4.19 ± 0.54 DnaX foci in the *dnaB*^+^ background. The loss of replisomes detected in the *dnaB8*(Ts) cells at the non-permissive temperature is consistent with the inability of this DnaB mutant to maintain processive replication at the non-permissive temperature.

The number of RecF and RecO foci per cell was comparable between the two *dnaB* backgrounds (RecF: 0.95 ± 0.07 in *dnaB*^+^, 1.32 ± 0.09 in *dnaB8*[Ts]; RecO: 0.14 ± 0.04 in *dnaB*^+^, 0.24 ± 0.07 in *dnaB8*[Ts], Figure 7C and D). Notably, the colocalization of RecF with replisomes was 1.5-fold higher in *dnaB8*(Ts) cells (36%) compared to *dnaB*^+^ cells (24%) (Supplementary Figure S15; chance colocalization ∼4% in both cases, see ‘Materials and methods’ section). Strikingly, the colocalization of RecO with replisomes increased from 5% in *dnaB8*(Ts) cells to 20% in *dnaB8*(Ts) cells (chance colocalization ∼4% in both cases, see ‘Materials and methods’ section). The enhanced colocalization of RecF and RecO with the replication forks may reflect the weaker helicase activity of DnaB8 compared to DnaB (53).

Irradiating with UV and increasing the temperature led to a reduction of RecF foci in *dnaB8*(Ts) cells (Figure 7C and Supplementary Figure S13) mirroring the previously observed reduction in DnaX foci (Figure 7B). By the 90 min time-point, *dnaB8*(Ts) cells contained on average only 0.40 ± 0.20 RecF foci per cell, compared with 2.71 ± 0.45 foci for *dnaB^+^* cells under the same conditions. A similar trend was observed for RecO foci (Figure 7D). By the 90 min time-point, *dnaB8*(Ts) cells contained on average only 0.26 ± 0.18 RecO foci per cell, compared with 0.6 ± 0.2 foci for *dnaB^+^* cells under the same conditions.

Our data demonstrate that UV irradiation leads to an increase in DnaX, RecF and RecO foci per cell in *dnaB*^+^ cells. Whereas RecF is often found at replisomes, most RecO molecules are resides at sites away from the replisome. Loss of replisomes is accompanied by an overall loss of RecF and RecO binding sites in cells. These findings lead us to suggest that whereas RecF may play a role at the replisome, RecO instead acts on substrates that are generated and left behind in the wake of the replisome—consistent with its proposed role in post-replicative gap repair.

## DISCUSSION

The epistatic relationship of the *recF, recO* and *recR* genes has led to the expectation that the proteins function together, perhaps forming a complex or forming multiple complexes in a temporal order at one location. Here, we examined this hypothesis by obtaining a high-resolution description of the spatial and temporal organization of RecF and RecO in cells following DNA damage. The evidence point to several key differences in the behaviour of RecF and RecO in cells. We found that the RecF protein spends most of its time near the centre of the nucleoid, often colocalizing with the replisome. This is true both before and after exposure to an UV challenge. The formation of RecF foci is strikingly dependent on DNA replication. In contrast, the RecO protein is usually found closer to the nucleoid/cell periphery, and RecO foci are rarely coincident with replisomes. The formation of RecO foci, however, is also dependent on DNA replication. In all of our experiments, RecF and RecO rarely colocalized with each other. The spatial and temporal properties of RecF and RecO foci imply differences in function. A distinction in function is also brought forward in phenotypic differences observed when cells are challenged with a broad range of DNA damaging agents. The results indicate that, irrespective of phenotypic similarities documented in earlier work, the RecF and RecO proteins have distinct functions in recombinational repair.

The RecOR complex is both necessary and sufficient for facilitating the nucleation of RecA filaments on SSB-coated ssDNA (7,11,35,45). The observation that RecO foci are usually found at some distance from replisomes might be consistent with a role in loading RecA protein at DNA post-replicative gaps and/or double-strand breaks spatially separated from replisomes. Additional support for a role in post-replicative gap repair comes from the observation that DNA replication is required for the UV-induced increase in the number of RecO foci. It is interesting to note that this same region of the cell in which RecO foci form plays host to large bundles of RecA, which are proposed to mediate double-strand break repair (6,70,71). The RecO localizations detected in our work may reflect intermediates formed during RecA loading during the DNA damage response. Unfortunately, it is not yet possible to simultaneously image fluorescently tagged RecO and RecA due to technical limitations; RecA is present at 10^5^–10^6^ molecules per cell (72–75) and thus bleed-through from fluorescently tagged RecA floods to the RecO channel. Alternative probes for RecA may alleviate this limitation in the future.

RecF can enhance RecOR-mediated RecA loading under certain (non-physiological) conditions *in vitro* (7,8), a clear role in this *in vivo* process has however not been demonstrated. We found no evidence supporting these observations under physiological conditions in live cells. RecF foci did not colocalize with RecO foci. Instead RecF frequently localized to replisomes, suggesting a potential RecF function at or near the replisome. Indeed, RecF foci are strongly dependent on the presence of active replication forks. A replisome-associated role for RecF is not an entirely new concept (76). RecF and RecR are required *in vivo* to recover replication after fork stalling, to prevent DNA degradation at stalled forks, and to complete ongoing replication (76). We also detected dimerization of RecF upon UV irradiation. RecF dimerization is required for fork recovery after UV irradiation in *E. coli* (67). The present results more directly tie RecF to a possible role at the replisome and are in line with the proposal that the effects of *recF* deletion on recombination may well stem from problems that arise at the replication fork (76).

## DATA AVAILABILITY

Example datasets and the script used to quantify the data have been made freely accessible (doi: 10.6084/m9.figshare.7409822).

## Supplementary Material

Supplementary Data
