# Supplementary material for: RecFOR epistasis group: RecF and RecO have distinct localizations and functions in Escherichia coli
Source: Nucleic Acids Res. 2019 Jan 18;47(6):2946–65. doi: 10.1093/nar/gkz003 (PMC6451095; doi:10.1093/nar/gkz003)

SUPPLEMENTARY FIGURES LEGENDS

Supplementary Figure S1. Functionality assays for fusion protein constructs.

(A) Growth curves of single-colour strains carrying the protein fusions RecF-YPet, RecF-mKate2, RecO-YPet, RecO-mKate2 or DnaX-YPet, as well as two-colour strains expressing RecO-mKate2 RecF-YPet, RecF-mKate2 DnaX-YPet or RecO-mKate2 DnaX-YPet. Wild-type, Δ*recF* and Δ*recO* strains were used as controls. Cells were grown in LB at 37°C over a period of 10h. Growth curves are averaged over biological quadruplets while error bars represent the standard deviation. No growth defects were observed.

(B) Fitness of single protein fusion constructs (RecF-YPet, RecF-mKate2, RecO-YPet, RecO-mKate2 or DnaX-YPet) and two-colour protein fusion construct (RecO-mKate2 RecF-YPet). For each construction, two growth competition assays were performed. First, the strain Ara+ of each protein fusion construct was compared to wild-type Ara- (EAW214). Second, the strain Ara- of each fusion protein construct was compare to the wild-type Ara+ (MG1655). The assay was started with 50% of each of the two strains in the population mixture. For each of the two competing strains, the percentage cell population was determined at t = 0, 24, 48 and 72h. Black lines represent Ara+ strains, red lines represent Ara- strains. Strains were indicated by symbol shape (wild-type: circle, RecF-YPet: upwards facing triangle, RecF-mKate2: downwards facing triangle, RecO-YPet: cross, RecO-mKate2: square, DnaX-YPet: diamond, RecO-mKate2 RecF-YPet: pentagon). Fusion protein constructs exhibit similar fitness to wild-type cells.

(C) UV survival assays. Cells grown in LB to exponential phase were serial diluted and spotted on two LB plates. One plate was exposed to 60 J/m^2 the other was used as a control for unexposed cells. Plates were incubated overnight at 37°C. Images show a representative experiment of independent triplicates. Strains expressing RecF-YPet, RecF-mKate2, RecO-YPet, RecO-mKate2, DnaX-YPet exhibit similar sensitivity to UV like wild-type. RecR fusions and DnaX-mKate2 fusion constructs were sensitised and showed similar sensitivity to Δ*recR* upon UV exposure.

(D) Bar graphs showing the fold induction of the SOS reporter fusion P*recN-lacZ* (pEAW362) in MG1655 (wild-type), EAW20 (Δ*recA*), EAW629 (Δ*recF*), EAW670 (*recF-YPet*), EAW779 (*recF-mKate2*), EAW114 (Δ*recO*), EAW814 (*recO-YPet*), EAW672 (*recO-mKate2*), JJC5945 (*dnaX-YPet*) and EAW676 (*recO-mKate2 recF-YPet*). Cells carrying P*recN-lacZ* were grown in LB_{Amp} at 37°C until reaching

exponential phase. Cultures were split; one subculture was treated with 0.25 μg/mL mitomycin C inducing the SOS reponse, the other was used as a control. After 2h of growth at 37°C, expression of P*recN-lacZ* was measured by the β-galactosidase assay. The SOS induction level (fold induction) was determined by dividing the β-galactosidase activity from the MMC treated culture by the β-galactosidase activity from the untreated control. The standard deviations across biological triplicates are indicated with error bars.

Supplementary Figure S2. Single-molecule imaging of RecF-mKate2 and RecO-YPet fusion constructs

Histograms showing the number of RecF-mKate2 and RecO-YPet foci per cell in response to UV irradiation. Bright-field images were used to determine the position of cells within different fields of view. The number of foci per cell were counted for each cell and plotted in a histogram. We plotted these histograms for the time-point before UV irradiation (0min) and several time-points following UV irradiation (10, 30, 60 and 90min). The mean over the number of foci per cell is depicted in each histogram for each time-point. The number of cells included in each histogram is also indicated as *n*.

Supplementary Figure S3. Burst acquisitions and analysis at 160 Wcm^{-2}.

(A) Experimental setup. Cells are loaded in a flow-cell and immobilised on a positively charged aminopropyl silane glass surface. Cells were images before UV exposure and 30-60min after UV exposure. UV exposure was conducted in flow-cells.

(B) Burst acquisition sequence. Movies of RecF-YPet or RecO-YPet were recorded. The movies contain 300 frames at 34ms recorded at continuous excitation.

(C) Exemplary average projection of one RecF-YPet movie. The average projection originates from one burst acquisition movie before UV exposure. The projection was made over 10 x 34ms. Two exemplary intensity trajectories are plotted showing RecF-YPet binding to DNA. Scale bar: 5 μm.

(D) Histrogram of bleaching step intensities. The histogram was fit with the Kernel density estimation function (orange line) to determine the bleaching step of a single YPet molecule with x_1 = 107.3, $x_2 = 2x_1$ and $x_3 = 3x_1$. The intensity of a single YPet molecule was used to determine the number of RecF-YPet and RecO-YPet molecules per focus in **Figure 3C**.

Supplementary Figure S4. Burst acquisitions and analysis at 16 Wcm^{-2}.

(A) Experimental setup. Cells are loaded in a flow-cell and immobilised on a positively charged aminopropyl silane glass surface. Cells were images before UV exposure and 30-60min after UV exposure. UV exposure was conducted in flow-cells.
(B) Burst acquisition sequence. Movies of RecF-YPet or RecO-YPet were recorded. The movies contain 300 frames at an exposure of 34ms.
(C) Exemplary average projections of one RecF-YPet movie before UV exposure and one movie after UV exposure. The average projection was made over 10 x 34ms. One exemplary intensity trajectories is plotted showing RecF-YPet binding to DNA before UV exposure and one trajectory that stems from a burst acquisition post UV exposure. Scale bar: 5 μm.
(D) Mean autocorrelation function is obtained from single autocorrelation function. Each autocorrelation function stems from single intensity trajectories of binding events.
(E) Determining components of autocorrelation functions. The autocorrelation function is plotted in black. The autocorrelation function has fast components which consist of noise, short-lived and transient binding events (light blue line). Slower components are fitted with a two exponential fit (dark blue line) which consist of medium and slow decorrelation events consistent with binding events.
(F) Components of the autocorrelation function are plotted in a bar graph. Slow, medium and fast components are indicated by shades of blue: slow (dark blue), medium (blue), fast (light blue). The error bars for slow and medium components were extracted from the fit error using the two exponential fit. The error bar from the fast components is equivalent to the standard error of the mean from the mean autocorrelation function at lag time 0s.

Supplementary Figure S5. RecF-YPet and RecO-YPet molecules per cell.
(A) Histogram of the number of RecF-YPet molecules per cell (blue) and RecO-YPet molecules per cell (grey) under normal growth conditions. The mean, standard error of the mean, standard deviation and number of cells (n = 71 for RecF-YPet; n = 98 for RecO-YPet) are depicted in each histogram.
(B) Histogram of the cell length for EAW670 (*recF-YPet*, blue) and EAW814 (*recO-YPet*, grey) under normal growth conditions. The mean, standard error of the mean, standard deviation and number of cells (n = 71 for RecF-YPet; n = 98 for RecO-YPet) are depicted in each histogram.

Supplementary Figure S6. Scatter plots of cell-size and fluorescence signal parameters from time-lapse imaging of cells expressing RecF-YPet (EAW670) or RecO-mKate2 (EAW672).White points indicate individual data-points, while blue-to-red contours indicate

frequencies of observations. Blue regions indicate regions with few data points and red regions indicate regions with a large number of data points. Frequencies were normalised at each sampled time-point to the maximum at each time-point.

(A) Distribution of cell length over time following UV irradiation directly after t = 0min. Fusion constructs grow into filaments at the same filamentation rate when exposed to UV light.

(B) Mean pixel intensity within cell boundaries. Changes in the mean cell intensity over time would indicate changes in the concentration of either RecF-YPet or RecO-mKate2. After UV exposure, mean cell intensities for cells expressing RecF-YPet or RecO-mKate2 stays constant indicating that the concentration of each protein stays constant as cells grow into filaments.

(C) Density of RecF-YPet and RecO-mKate2 foci per cell, measured as the number of foci per cell area in μm^2. The foci density for RecF-YPet and RecO-mKate2 stays relatively constant in response to UV damage. The density of RecF-YPet foci has a slight dip at 30min after UV irradiation exactly when nucleoids compact and colocalisation with replisomes is increased. The foci density of RecF-YPet foci is overall higher than the density of RecO-mKate2 foci since cells contain more RecF-YPet foci per cell then RecO-mKate2 foci (**Figure 2D**).

Supplementary Figure S7. Autocorrelation analysis of simulated rapid-acquisition data. To determine what effect the dimerization of a protein would have on the autocorrelation analysis of rapid-acquisition movies, simulated data were generated. Intensity versus time trajectories were generated for two types of complexes: one containing one molecule per complex (representing RecF-YPet), and one containing two molecules per complex (representing $[\text{RecF-YPet}]_2$). Custom Matlab code (**Appendix PDF**) was used to simulate a scenario in which each complex binds to and dissociates from DNA, while the pool of available fluorescent proteins photobleaches. Signal is only generated when a complex is bound, the complex contains a molecule, and the molecule has not yet photobleached, analogous to focus formation in the experimental data. User-defined parameters are described within the code. For the displayed simulation the parameters were set as follows: *frames* = 100; *signal* = 1; *bkg* = 3; *max_sites_mol1* = 1; *max_sites_mol2* = 2; *complexes* = 1000; *mol_per_complex* = 10; *fracunbndt0* = 0; *Kon_complex* = 0.00001; *Koff_complex* = 0.03; *StoN_mol1* = 3; *Tbleach_mol1* = 50; *Kon_mol1 = 0.00001*; *Koff_mol1 = 0.00001*; *StoN_mol2* = 3; *Tbleach_mol2* = 50; *Kon_mol2* = 0.00001; *Koff_mol2* = 0.00001. Autocorrelation functions were calculated as for the experimental data (**Materials and Methods**). Comparing the autocorrelation function for the simulated data (above) against that for the experimental data (**Figure 3D**) it is clear that dimerization of RecF (**Figure 3C**)

would be sufficient to explain the increase in autocorrelation observed after UV-irradiation of cells.

Supplementary Figure S8. Time-lapse images of RecF-YPet in wild type, Δ*recA*, Δ*recR* and Δ*recO*. Time-lapse images are shown at 0, 30, 60, 90 and 120min after UV irradiation. Cell outlines indicate the boundaries of single cells. In wild type cells, the number of RecF-YPet foci increases as cells filament. In a Δ*recA* background, cells do not grow into filaments and lose their RecF-YPet foci in response to UV damage. In a Δ*recR* background, cells either do not grow into filaments or filament slower than wild type cells. Cells that do not filament lose their RecF-YPet foci, whereas, cells that slowly filament have some foci. In a Δ*recO* background, cells either do not grow into filaments or filament slower than wild type cells. All Δ*recO* cells contain RecF-YPet foci. Scale bar: 5 μm.

Supplementary Figure S9. Time-lapse images of RecO-mKate2 in wild type, Δ*recA*, Δ*recR* and Δ*recF*. Time-lapse images are shown at 0, 30, 60, 90 and 120min after UV irradiation. Cell outlines indicate the boundaries of single cells. In wild type cells, the number of RecO-mKate2 foci increases as cells filament. In a Δ*recA* background, cells do not grow into filaments and lose their RecO-mKate2 foci in response to UV damage. In a Δ*recR* background, cells either do not grow into filaments or filament slower than wild type cells. All Δ*recR* cells contain RecO-mKate2 foci. In a Δ*recF* background, cells either do not grow into filaments or filament slower than wild type cells. All Δ*recF* cells contain RecO-mKate2 foci. Scale bar: 5 μm.

Supplementary Figure S10. Scatter plots of cell length over time from time-lapse imaging of cells expressing RecF-YPet (EAW670) or RecO-mKate2 (EAW672) in comparison to fusion constructs lacking *recR* (SSH068, EAW697), *recA* (SSH070, SSH067) and either *recO* (EAW824) or *recF* (EAW822). Cells were exposed to a single UV dose directly after t = 0min. All contour plots have white points indicating individual data-points, while blue-to-red contours illustrate frequencies of observations. Blue regions indicate regions with few data points and red regions indicate regions with a large number of data points. Frequencies were normalised at each sampled time-point to the maximum at each time-point.

(A) Fusion constructs that do have no deletion background grow into filaments at the same filamentation rate when exposed to UV light. Same figure as **Supplementary Figure S6**.

(B) Cells expressing RecF-YPet while lacking *recO* have two populations of cells post UV exposure, one does not grow while the other slowly filaments. In contrast, cells

expressing RecO-mKate2 mostly grow into filaments at a slower rate than wild type cells.

(C) Cells lacking *recR* and expressing either RecF-YPet or RecO-mKate2 have two populations of cells post UV exposure, one does not grow while the other slowly filaments. This behaviour is similar to cells lacking *recO* (**Supplementary Figure S10B**).

(D) Cells lacking *recA* and expressing either RecF-YPet or RecO-mKate2 either divide once and then do not grow or do not grow at all post UV treatment.

Supplementary Figure S11. Histograms of the number of RecF-YPet foci in wild type cells and cells lacking *recA*, *recR* or *recO*. Bright-field images were used to determine the position of cells within different fields of view. The number of foci per cell were counted for each cell and plotted in a histogram. We plotted these histograms for the time-point before UV irradiation (0min) and several time-points following UV irradiation (10, 30, 60 and 90min). The mean over the number of foci per cell is depicted in each histogram for each time-point. The number of cells included in each histogram is also indicated as *n*. Wild-type cells contain more foci per cell as cells grow into filaments. Cells lacking *recA* lose their RecF-YPet foci post UV exposure. Cells lacking *recR* contain less RecF-YPet foci under normal growth conditions (see **Supplementary Figure S8** for time-lapse images). Post UV, cells either lose their RecF foci or have some RecF foci. Cells lacking *recO* however slightly increase the number of RecF foci until 90min after UV exposure.

Supplementary Figure S12. Histograms of the number of RecO-mkate foci in wild type cells and cells lacking *recA*, *recR* or *recF*. Bright-field images were used to determine the position of cells within different fields of view. The number of foci per cell were counted for each cell and plotted in a histogram. We plotted these histograms for the time-point before UV irradiation (0min) and several time-points following UV irradiation (10, 30, 60 and 90min). The mean over the number of foci per cell is depicted in each histogram for each time-point. The number of cells included in each histogram is also indicated as *n*. Corresponding time-lapse images are shown in **Supplementary Figure S9**. Upon UV exposure, more wild type cells contain RecO-mKate2 foci while some still have zero foci. Most cells lacking *recA* contain no foci after UV exposure. Cells lacking *recR* or *recF* however have more RecO foci after a single UV dose.

Supplementary Figure S13. Experimental design and images (unfiltered and filtered) of CJH0015 (*recF-mKate2 dnaX-YPet dnaB*$^{+}$), EAW762 (*recO-mKate2 dnaX-YPet dnaB*$^{+}$), SSH114 (*recF-mKate2 dnaX-YPet dnaB8*(Ts)) and SSH115 (*recO-mKate2 dnaX-YPet dnaB8*(Ts)) at 0, 30 and 90min.

(A) Experimental design. First image is taken at 30˚C (0min) when no UV image is yet induced. Then, the temperature is ramped up to 42˚C. UV damage is induced at 3-4min. 42˚C are reached at 5min and hold until the end of the experiment, at 120min.

(B) Images of *recF-mKate2 dnaX-YPet dnaB*$^{+}$. Upper panel: DnaX-YPet signal, raw images. Middle panel: RecF-mKate2 signal, raw images. Lower panel: Merged images of RecF-mKate2 (magenta signal) and DnaX-YPet (green signal) are shown before UV irradiation, at 30˚C, and after UV irradiation, at 42˚C (30min and 90min). Scale bar: 5 μm.

(C) Images of *recF-mKate2 dnaX-YPet dnaB8*(Ts). Upper panel: DnaX-YPet signal, raw images. Middle panel: RecF-mKate2 signal, raw images. Lower panel: Merged images of RecF-mKate2 (magenta signal) and DnaX-YPet (green signal) are shown before UV irradiation, at 30˚C, and after UV irradiation, at 42˚C (30min and 90min). Scale bar: 5 μm.

(D) Images of *recO-mKate2 dnaX-YPet dnaB*$^{+}$. Upper panel: DnaX-YPet signal, raw images. Middle panel: RecF-mKate2 signal, raw images. Lower panel: Merged images of RecF-mKate2 (magenta signal) and DnaX-YPet (green signal) are shown before UV irradiation, at 30˚C, and after UV irradiation, at 42˚C (30min and 90min). Scale bar: 5 μm.

(E) Images of *recO-mKate2 dnaX-YPet dnaB8*(Ts). Upper panel: DnaX-YPet signal, raw images. Middle panel: RecF-mKate2 signal, raw images. Lower panel: Merged images of RecF-mKate2 (magenta signal) and DnaX-YPet (green signal) are shown before UV irradiation, at 30˚C, and after UV irradiation, at 42˚C (30min and 90min). Scale bar: 5 μm.

Supplementary Figure S14. The non-permissive temperature of cells carrying the dnaB8(Ts) allele is 42°C. Spot plate dilution assays of MG1655 (wild-type), HG362 (*dnaB8*[Ts]), CJH0015 (*recF-mKate2 dnaX-YPet dnaB*$^{+}$), SSH114 (*recF-mKate2 dnaX-YPet dnaB8*[Ts]), EAW762 (*recO-mKate2 dnaX-YPet dnaB*$^{+}$) and SSH115 (*recO-mKate2 dnaX-YPet dnaB8*[Ts]). Cells grown to exponential phase (OD_{600} ~0.2) were serial diluted to the dilution 10^{-5}. Serial dilutions were spotted on LB agar. Plates were incubated overnight either at 37°C or 42°C. Images show a representative experiment of independent duplicates.

Supplementary Figure S15. Experimental design and colocalisation measurements of CJH0015 (*recF-mKate2 dnaX-YPet dnaB*$^{+}$), EAW762 (*recO-mKate2 dnaX-YPet dnaB*$^{+}$), SSH114 (*recF-mKate2 dnaX-YPet dnaB8*(Ts)) and SSH115 (*recO-mKate2 dnaX-YPet dnaB8*(Ts)) at 0, 30 and 90min. Colocalisation was measured over >300 cells.

(A) Experimental design. First image is taken at 30˚C (0min) when no UV image is yet induced. Then, the temperature is ramped up to 42˚C. UV damage is induced at 3-4min. 42˚C are reached at 5min and hold until the end of the experiment, at 120min.

(B) Colocalisation measurement of RecF-mKate2 with DnaX-YPet in *dnaB*$^{+}$. The percentage of RecF-mKate2 foci that contain a DnaX-YPet focus is plotted as a magenta line plot over 120min at intervals of 10min. Similarly, the colocalisation of DnaX-YPet with RecF-mKate2 is plotted as a green line plot.

(C) Colocalisation measurement of RecF-mKate2 with DnaX-YPet in *dnaB8*(Ts). The percentage of RecF-mKate2 foci that contain a DnaX-YPet focus is plotted as a magenta line plot over 120min at intervals of 10min. Similarly, the colocalisation of DnaX-YPet with RecF-mKate2 is plotted as a green line plot.

(D) Colocalisation measurement of RecO-mKate2 with DnaX-YPet in *dnaB*$^{+}$. The percentage of RecO-mKate2 foci that contain a DnaX-YPet focus is plotted as a magenta line plot over 120min at intervals of 10min. Similarly, the colocalisation of DnaX-YPet with RecO-mKate2 is plotted as a green line plot.

(E) Colocalisation measurement of RecO-mKate2 with DnaX-YPet in *dnaB8*(Ts). The percentage of RecO-mKate2 foci that contain a DnaX-YPet focus is plotted as a magenta line plot over 120min at intervals of 10min. Similarly, the colocalisation of DnaX-YPet with RecO-mKate2 is plotted as a green line plot.

Supplementary Figure S16. Colocalisation measurements of CJH0015 (*recF-mKate2 dnaX-YPet dnaB*$^{+}$) following ciprofloxacin treatment (30 ng/mL). Colocalisation was measured over >300 cells. The percentage of RecF-mKate2 foci that contain a DnaX-YPet focus is plotted as a magenta line plot over 180min at intervals of 10min. Similarly, the colocalisation of DnaX-YPet with RecF-mKate2 is plotted as a green line plot.

Supplementary Figure S17. Time-lapse data of cells carrying a pBAD plasmid to express either YPet or mKate2. Expression is induced at low levels with $5{\cdot}10^{-3}$% L-arabinose. Cells were exposed to a UV dose of 10 $J{\cdot}m^{-2}$ directly after t = 0min. Images are shown at 0, 30, 60 and 90min. Upper panel: signal of cells expressing YPet. The fluorescent protein YPet does not form foci after UV exposure. Bottom panel: signal of cells expressing mKate2. The fluorescent protein mKate2 does show some diffusive signal after UV exposure, however, no foci. Scale bar: 5 μm.

MATLAB CODE

```
%%%%%%%%%%%%%%%%%%%%%%%%%%%%%%%%%%%%%%%%%%%%%%%%%%%%%%%%%%%%%%%%%%%%%%%%%%%%%%%%%%%%%%%%%%%%%%%%%%%%%%%%%%%%%%%%%%%%%%%%%%%%%%%%%%%%%%%%%%%%%%%%%%
%%%%%%%%%%%%%%%%%%%%%%
%%%Thank you to Dr Jason Otterstrom for providing the nucleus of this code
%%%%%%%%%%%%%%%%%%%%%%%%%%%%%%%%%%%%%%%%%%%%%%%%%%%%%%%%%%%%%%%%%%%%%%%%%%%%%%%%%%%%%%%%%%%%%%%%%%%%%%%%%%%%%%%%%%%%%%%%%%%%%%%%%%%%%%%%%%%%%%%%%%
%%%%%%%%%%%%%%%%%%%%%%

% Initialise
clear all

%%% Input parameters %%%
frames = 100; % number of time steps
Signal = 1; % intensity per molecule
bkg = 3; % signal−to−background ratio

max  sites  mol1 = 1; % maximum number of binding sites for molecule 1 within the complex
max  sites  mol2 = 2; % maximum number of binding sites for molecule 2 within the complex
complexes = 1000; % number of complexes to simulate
mol  per  complex = 10; % number of molecules available to bind (or not) per complex
fracunbndt0 = 0; % fraction of complexes that have no molecule bound at time = 0

Kon  complex = 0.00001; % Kon in inverse frames; complex binding to DNA
Koff  complex = 0.03; % Koff; in inverse frames; complex dissociating from DNA

StoN  mol1 = 3; % signal−to−noise ratio for molecule 1
Tbleach  mol1 = 50; % Time constant Tau for photobleaching of molecule 1
Kon  mol1 = 0.00001; % Kon in inverse frames; molecule 1 binding to complex
Koff  mol1 = 0.00001; % Koff; in inverse frames; molecule 1 dissociating from complex

StoN  mol2 = 3; % signal−to−noise ratio for molecule 2
Tbleach  mol2 = 50; % Time constant Tau for photobleaching of molecule 2
Kon  mol2 = 0.00001; % Kon in inverse frames; molecule 2 binding to complex
Koff  mol2 = 0.00001; % Koff; in inverse frames; molecule 2 dissociating from complex
%%%%%%%%%%%%%%%%%%%%%%%%%%%%%%%%%%%%%%%%%%%%%%%%%%%%%%%%%%%%%%%%%%%%%%%%%%%%%%%%%%%%%%%%%%%%%%%%%%%%%%%%%%%%%%%%%%%%%%%%%%%%%%%%%%%%%%%%%%%%%%%%%%
%%%%%%%%%%%%%%%%%%%%%%

% Generate binding/bleaching trajectories for molecules 1 and 2
Nmolec = complexes*mol  per  complex;

mol1  signal = zeros(frames,Nmolec);
mol2  signal = zeros(frames,Nmolec);

for m = 1:Nmolec
    mol1  signal(:,m) = Mol  binding(max  sites  mol1,frames,1,Kon  mol1,Koff  mol1,
   Tbleach  mol1,0,StoN  mol1);
    mol2  signal(:,m) = Mol  binding(max  sites  mol2,frames,1,Kon  mol2,Koff  mol2,
    Tbleach  mol2,0,StoN  mol2);
end

% Set mean and width for distributions of Kon and Koff values for complex binding to
DNA.
KonVal =     [1 0.1]*  Kon  complex;   % units are 'frames^−1'
KoffVal =    [1 0.1]*  Koff  complex;

% Determine the Kon and Koff values for every frame at every binding site
Kon = norminv(rand(frames,complexes),KonVal(1), KonVal(2));
Koff = norminv(rand(frames,complexes),KoffVal(1),KoffVal(2));

% Set random selection array
SelArray = rand(frames,complexes);

% Define an ID for each molecule having a bleaching traj
moleculeID = [zeros(Nmolec,1), rand(Nmolec,1)];

% Set binding trajectories
bound = zeros(frames,complexes);

% initialize the bound state
f = 1;
```

```
bound(f,:) = rand(1,complexes);
bound(f,bound(f,:) > fracunbndt0) = 1;
bound(f,bound(f,:) <= fracunbndt0) = 0;
idxbd = find(bound(f,:)==1);
if ~isempty(idxbd)
    [~,idx] =  min(abs(rand()-moleculeID(:,2)));
    bound(f,idxbd(1)) = idx;
    moleculeID(idx(1),1) = 1;
    for i = 2:size(idxbd,2)
        s = idxbd(i);
        molsel = 0;
        while molsel == 0
            [~,idx] = min(abs(rand()-moleculeID(:,2)));
            if moleculeID(idx,1) == 0
                molsel = 1;
                bound(f,s) = idx;
                moleculeID(idx,1) = 1;
            end
        end
    end
end

% Set binding state for all subsequent frames

for f = 2:frames

    idxbd = find(bound(f-1,:) ~= 0); % bound sites
    idxun = find(bound(f-1,:) == 0); % unbound sites

    % with bound sites, determine if they stay bound, or unbind in the
    % current frame
    for i = 1:size(idxbd,2)

        s = idxbd(i);

        Punbind = Koff(f,s)*exp(-Koff(f,s));
        %         Pstay = 1-Punbind;
        if SelArray(f,s) <= Punbind % molecule unbinds
            bound(f,s) = 0;
            moleculeID(bound(f-1,s),1) = 0;
        else % molecule stays bound
            bound(f,s) = bound(f-1,s);
        end
    end

    % with unbound sites, determine if they stay unbound, or bind a new
    % molecule
    for i = 1:size(idxun,2)

        s = idxun(i);
        Pbind = Kon(f,s)*exp(-Kon(f,s));
        if SelArray(f,s) <= Pbind
            molsel = 0;
            while molsel == 0
                [~,idx] = min(abs(rand()-moleculeID(:,2)));
                if moleculeID(idx,1) == 0
                    molsel = 1;
                    bound(f,s) = idx;
                    moleculeID(idx,1) = 1;
                end
            end
        end
    end
end

traj_bound = bound;
traj_bound(traj_bound>0)=Signal;

% Combine bound states and molecule bleaching to define signal trajectory
traj_signal_mol1 = norminv(rand(frames,complexes),0,Signal/bkg);
traj_signal_mol2 = norminv(rand(frames,complexes),0,Signal/bkg);
```

```
for f = 1:frames
    for s = 1:complexes
        molsel = bound(f,s);
        if molsel~=0
            traj  signal  mol1(f,s) = mol1  signal(f,s);
            traj  signal  mol2(f,s) = mol2  signal(f,s);
        end
    end
end

% Plot example trajectories for mol1 (top row) and mol2 (middle row)
for i = 1:5
    subplot(3,6,i)
    plot(traj  signal  mol1(:,i),'k')
end

for i = 7:11
    subplot(3,6,i)
    plot(traj  signal  mol2(:,(i−6)),'r')
end

% Plot overlaid example trajectories for mol1 and mol2 (bottom row)
for i = 13:17
    subplot(3,6,i)
    plot(1:frames,traj  signal  mol1(:,(i−12)),'k',1:frames,traj  signal  mol2(:,(i−
12)),'r')
end

% Plot mean autocorrelation functions for mol1 (top row), mol2 (middle row,
% and cross−correlation for mol1/mol2 (bottom row). This curve will be
% corrected for correlated photobleaching effects using the ccf for randomised set of
% mol1 and mol2 trajectories



for i = 1:complexes
    acf  mol1(:,i) = xcorr(traj  signal  mol1(:,i),'coeff');
    acf  mol2(:,i) = xcorr(traj  signal  mol2(:,i),'coeff');
    ccf  mol12(:,i) = xcorr(traj  signal  mol1(:,i),traj  signal  mol2(:,i),'coeff');
end

% generate set of mol2 trajectories with randomised order, 100 times

index = randperm(complexes);
traj  signal  mol2  rand = traj  signal  mol2(:,index);

for j = 1:100;
    index = randperm(complexes);
    traj  signal  mol2  rand = traj  signal  mol2(:,index);
    for i = 1:complexes
        ccf  mol12  rand(:,i,j) = xcorr(traj  signal  mol1(:,i),traj  signal  mol2  rand(:,i),'coeff');
    end
    clearvars index traj  signal  mol2  rand
end

% crop acfs and take mean

acf mol1 = acf mol1(frames:end,:);
acf  mol2 = acf  mol2(frames:end,:);
mean  acf  mol1 = mean(acf  mol1,2);
mean  acf  mol2 = mean(acf  mol2,2);
mean  ccf  mol12 = mean(ccf  mol12,2);
mean  ccf  mol12  rand = mean(ccf  mol12  rand,2);
mean  ccf  mol12  rand = mean(mean  ccf  mol12  rand,3);

subplot(3,6,6)
plot(0:frames−1,mean  acf  mol1,'k');
axis([0 frames −0.1 1.1])

subplot(3,6,12)
```

```
plot(0:frames−1,mean_acf_mol2,'r');
axis([0 frames −0.1 1.1])

subplot(3,6,18)
plot(−frames+1:frames−1,mean_ccf_mol12−mean_ccf_mol12_rand,'b');
axis([−frames frames −1.1 1.1])


function mol_signal = Mol_binding(Nsites,Nframes,Signal,Kon_in,Koff_in,Tbleach_in,
fracunbndt0,StoN)

%%% output arrays: structure => column = site, row = frame
%%%
%%% bound − array that keeps track of which site is bound by which molecule
%%%     in each frame
%%% molecule − array that keeps track of the bleaching state for every
%%%     molecule available for binding
%%% traj_bound − array similar to 'bound' but only with ones and zeros,
%%%     ones indicating a bound state
%%% traj_signal − similar to traj_bound but with added noise and a signal
%%%     hight equal to 'Signal' given at input

KonVal =     [1 0.1]*_Kon_in;   % units are 'frames';
KoffVal =    [1 0.1]*_Koff_in;
TbleachVal = [1 0.1]*_Tbleach_in;

Nmolec = Nsites*10;
%
% determine the kon and koff rates for every frame at every site
Kon = norminv(rand(Nframes,Nsites),KonVal(1), KonVal(2));
Koff = norminv(rand(Nframes,Nsites),KoffVal(1),KoffVal(2));

% set random selection array
SelArray = rand(Nframes,Nsites);

% Set photobleaching trajectories
% determine the bleaching constant for every molecule
Tbleach = norminv(rand(1,Nmolec),TbleachVal(1),TbleachVal(2));
bleachframe = ceil(expinv(rand(1,Nmolec),Tbleach));
molecule = zeros(Nframes,Nmolec);

% define the bleaching trajectory for every molecule available for binding
for m = 1:Nmolec
   if bleachframe(m)−1 <= Nframes
      molecule(1:bleachframe(m)−1,m) = Signal;
   else
      molecule(:,m) = Signal;
   end
end

% define an ID for each molecule having a bleaching traj
moleculeID = [zeros(Nmolec,1), rand(Nmolec,1)];

% Set binding trajectories
bound = zeros(Nframes,Nsites);

% initialize the bound state
f = 1;
bound(f,:) = rand(1,Nsites);
bound(f,bound(f,:) > fracunbndt0) = 1;
bound(f,bound(f,:) <= fracunbndt0) = 0;
idxbd = find(bound(f,:)==1);

if ~isempty(idxbd)
   [~,idx] =  min(abs(rand()−moleculeID(:,2)));
   bound(f,idxbd(1)) = idx;
   moleculeID(idx(1),1) = 1;
   for i = 2:size(idxbd,2)
      s = idxbd(i);
      molsel = 0;
      while molsel == 0
```

```
            [~,idx] = min(abs(rand()-moleculeID(:,2)));
            if moleculeID(idx,1) == 0
                molsel = 1;
                bound(f,s) = idx;
                moleculeID(idx,1) = 1;
            end
        end
    end
end

% Set binding state for all subsequent frames
for f = 2:Nframes

    idxbd = find(bound(f-1,:) ~= 0); % bound sites
    idxun = find(bound(f-1,:) == 0); % unbound sites

    % with bound sites, determine if they stay bound, or unbind in the
    % current frame
    for i = 1:size(idxbd,2)
        s = idxbd(i);
        Punbind = Koff(f,s)*exp(-Koff(f,s));
        %         Pstay = 1-Punbind;
        if SelArray(f,s) <= Punbind % molecule unbinds
            bound(f,s) = 0;
            moleculeID(bound(f-1,s),1) = 0;
        else % molecule stays bound
            bound(f,s) = bound(f-1,s);
        end
    end

    % with unbound sites, determine if they stay unbound, or bind a new
    % molecule
    for i = 1:size(idxun,2)
        s = idxun(i);
        Pbind = Kon(f,s)*exp(-Kon(f,s));
        if SelArray(f,s) <= Pbind
            molsel = 0;
            while molsel == 0
                [~,idx] = min(abs(rand()-moleculeID(:,2)));
                if moleculeID(idx,1) == 0
                    molsel = 1;
                    bound(f,s) = idx;
                    moleculeID(idx,1) = 1;
                end
            end
        end
    end
end


traj_bound = bound;
traj_bound(traj_bound>0)=Signal;


% initialize the signal trajectory as only noise around zero
%traj_signal = norminv(rand(Nframes,Nsites),0,Signal/StoN);
traj_signal = zeros(Nframes,Nsites);
% if a site has a non-bleached molecule bound, increase the signal
for f = 1:Nframes
    for s = 1:Nsites
        molsel = bound(f,s);
        if molsel~=0 && molecule(f,molsel)~= 0
            traj_signal(f,s) = Signal + traj_signal(f,s);
        end
    end
end

avbleach = mean(molecule,2);
nfftB = 2^nextpow2(2*size(avbleach,1)-1);
r_B = ifft( fft(avbleach,nfftB) .* conj(fft(avbleach,nfftB)) );
```

```
avsig = mean(traj  signal,2);
nfftS = 2^nextpow2(2*size(avsig,1)−1);
r  S = ifft( fft(avsig,nfftS) .* conj(fft(avsig,nfftS)) );

mol  signal  temp=sum(traj  signal,2);

for i = 1:Nframes
    noise = mean(norminv(rand((mol  signal  temp(i)+1),1),0,Signal/StoN));
        mol  signal(i) = mol  signal  temp(i) + noise;
    clearvars noise
end
```

1

Supplementary Figure S1

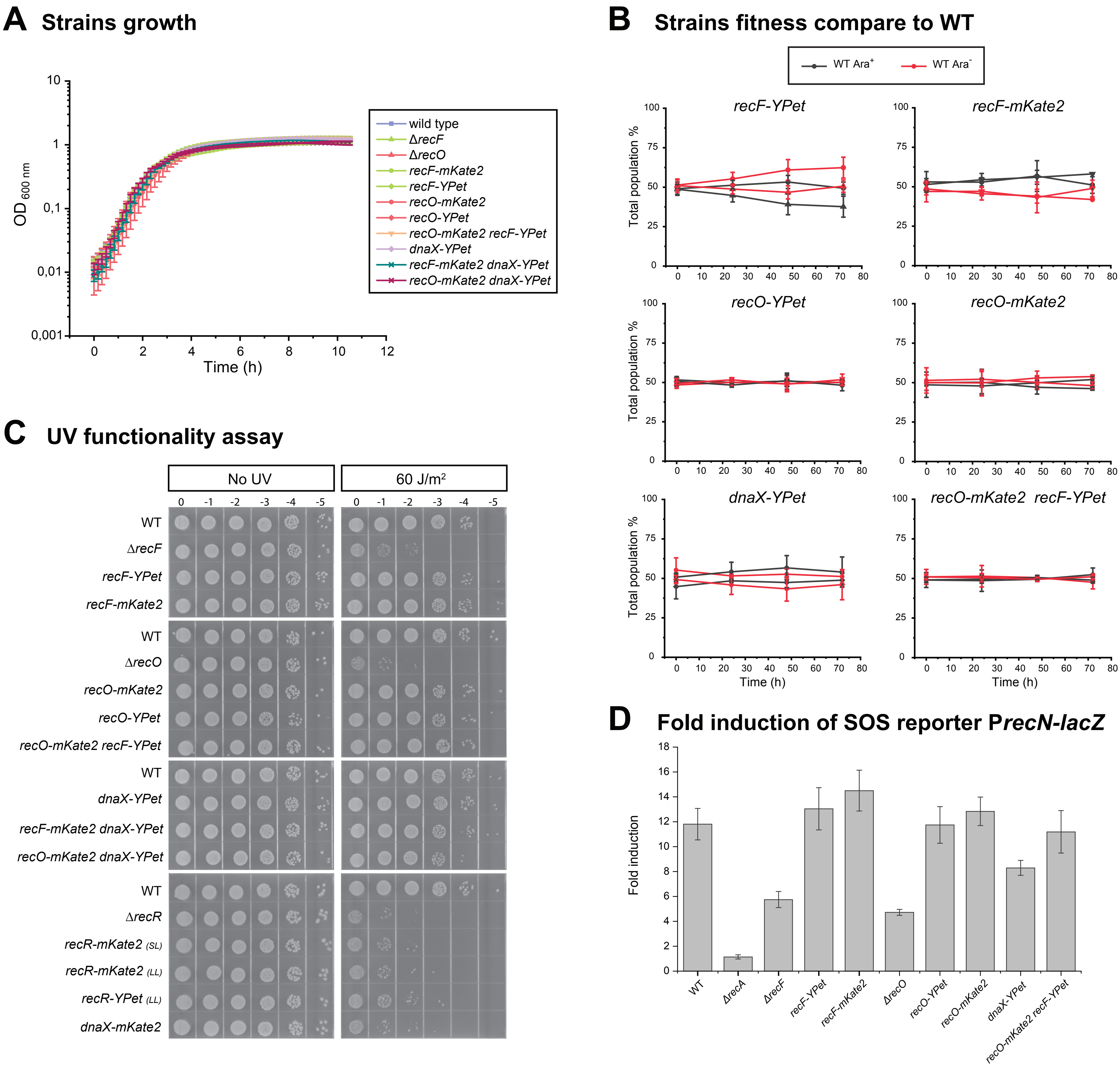

Supplementary Figure S2

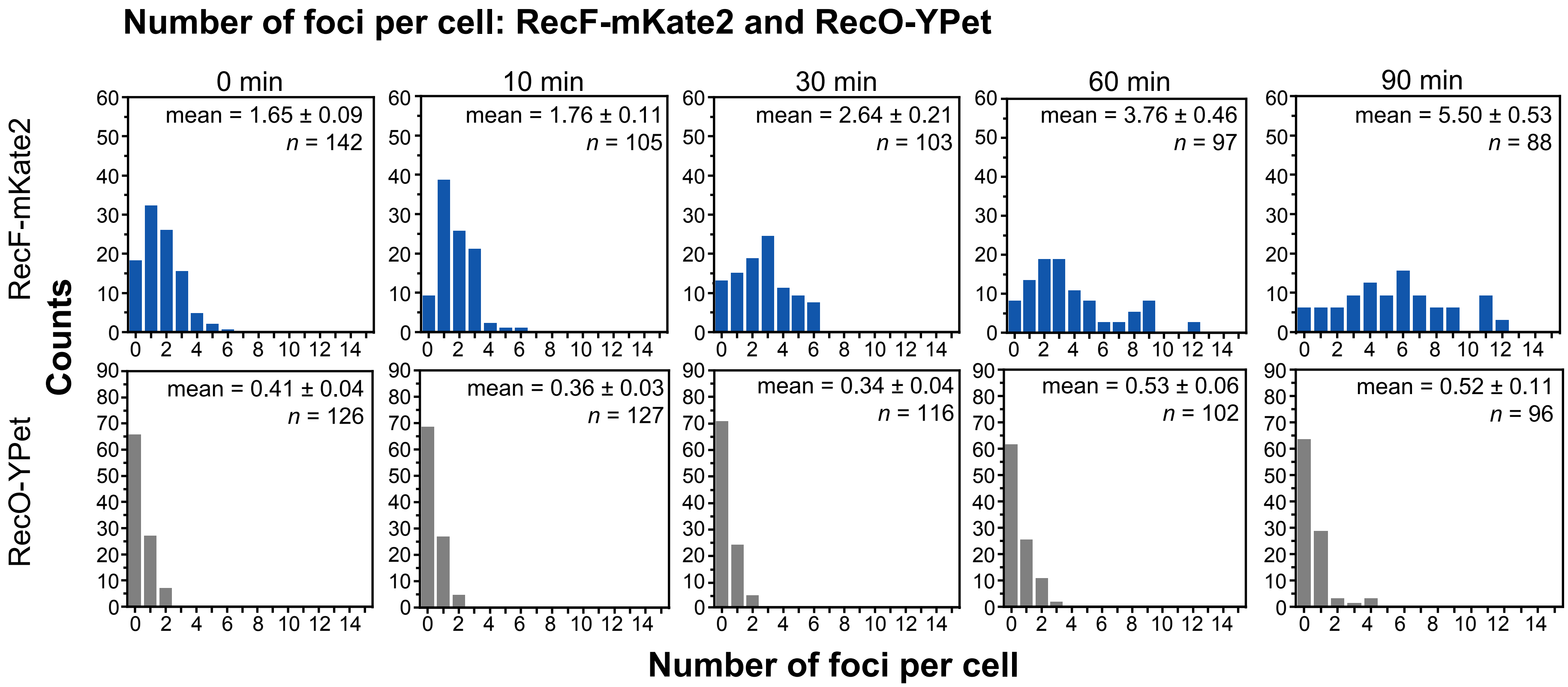

Supplementary Figure S3

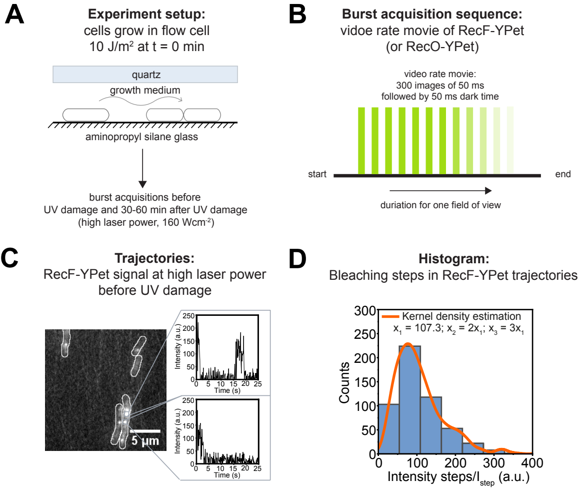

Supplementary Figure S4

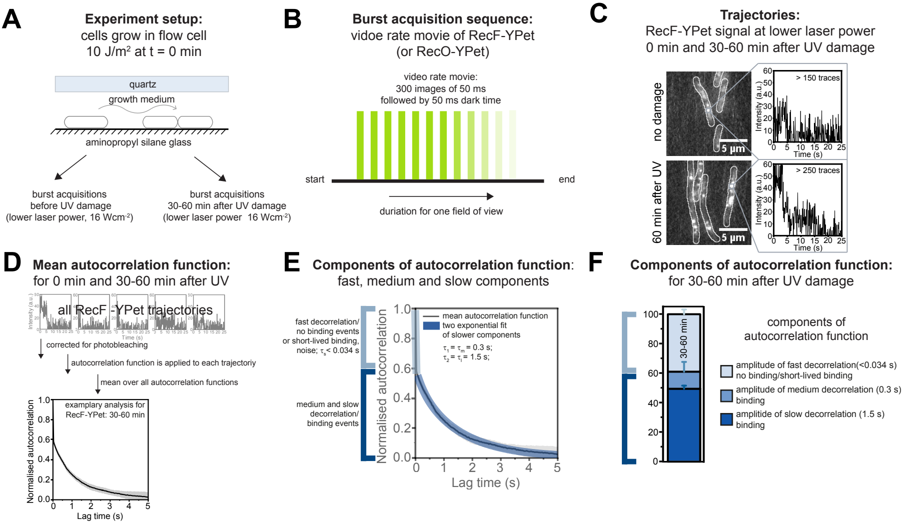

Supplementary Figure S5

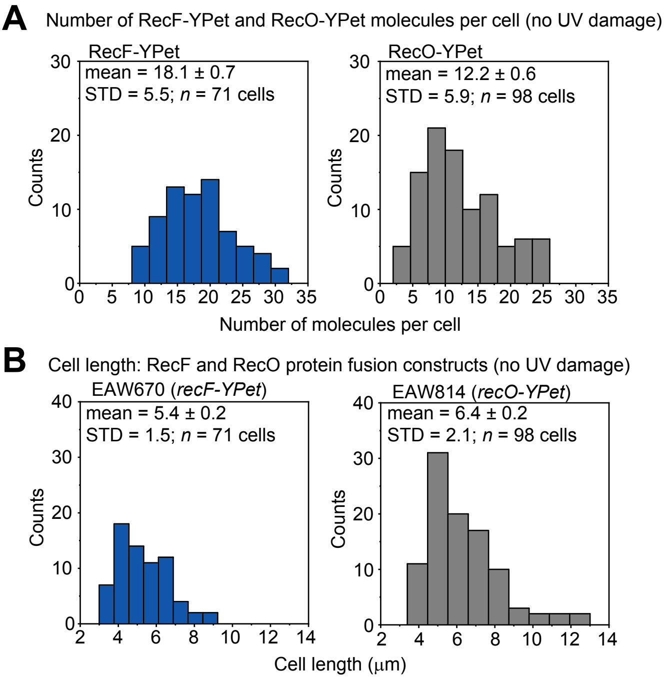

Supplementary Figure S6

A Cell length vs time

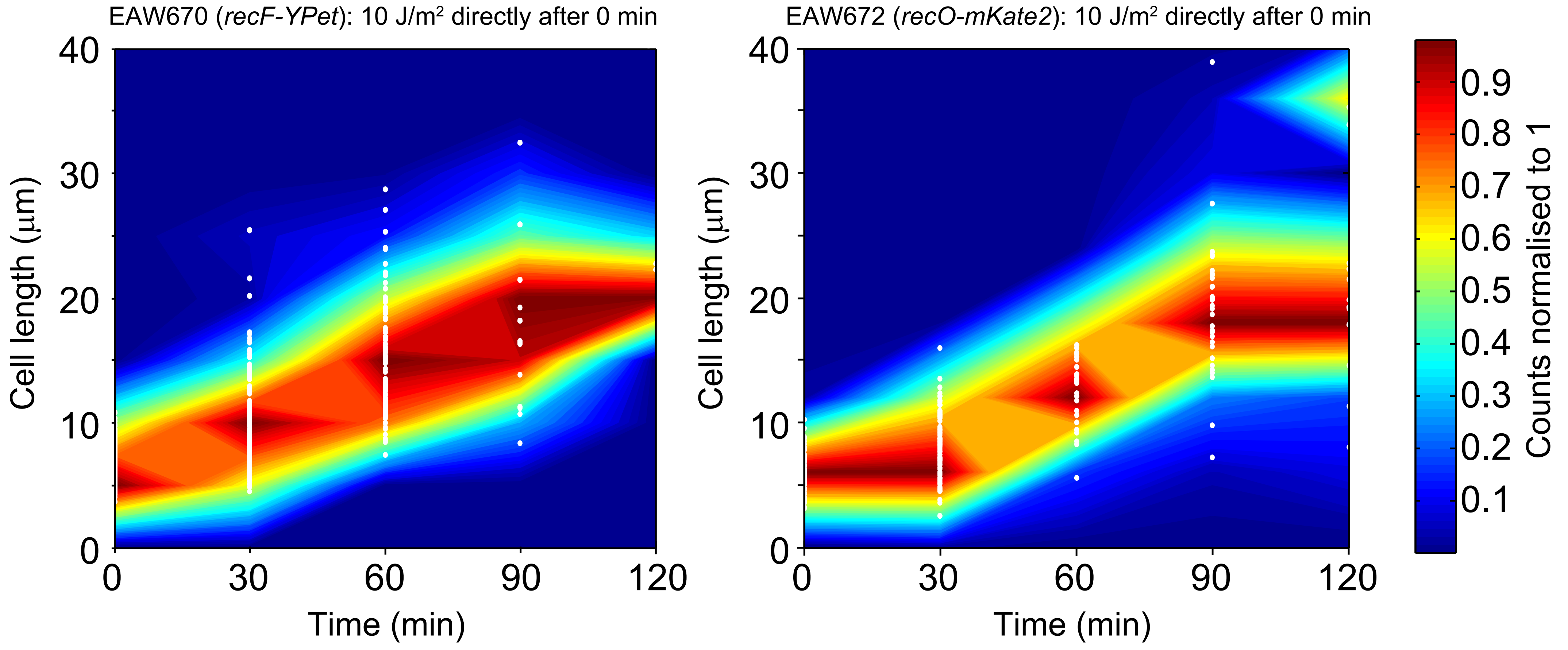

B Mean cell intensity/protein concentration vs time

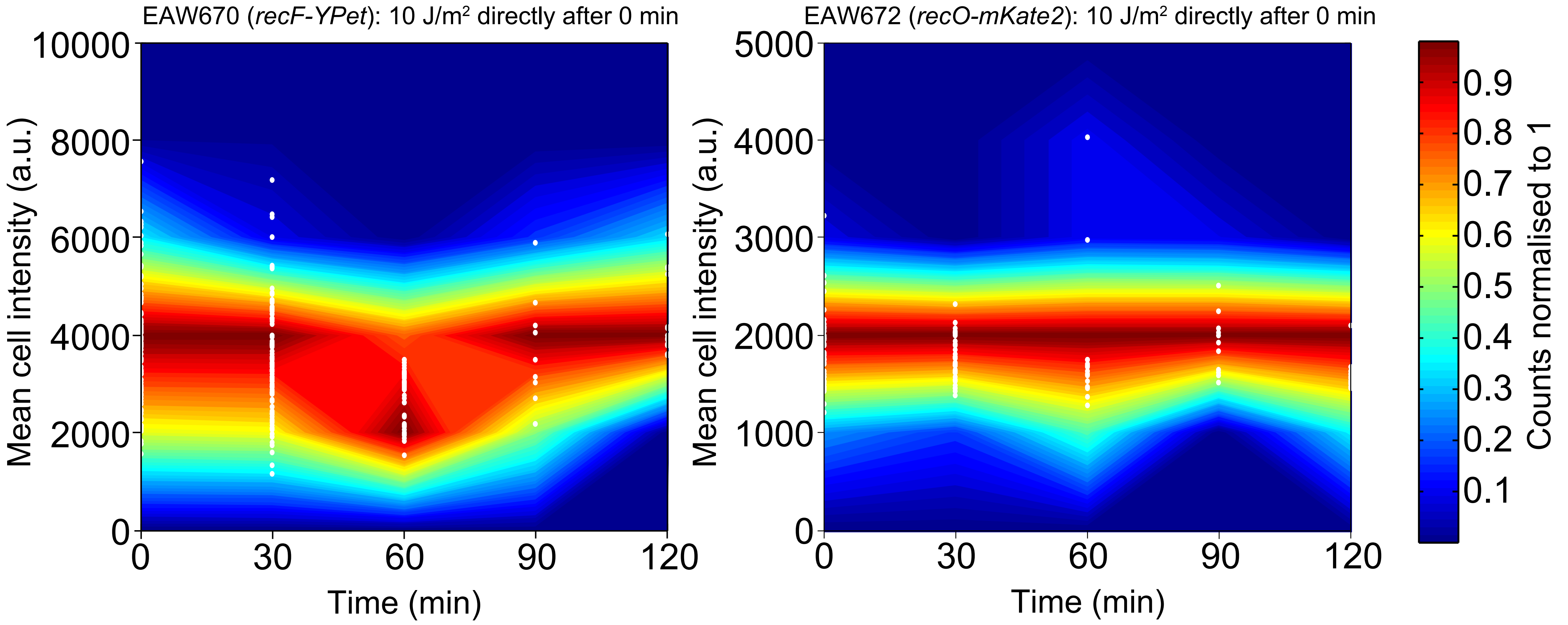

C Density of foci vs time

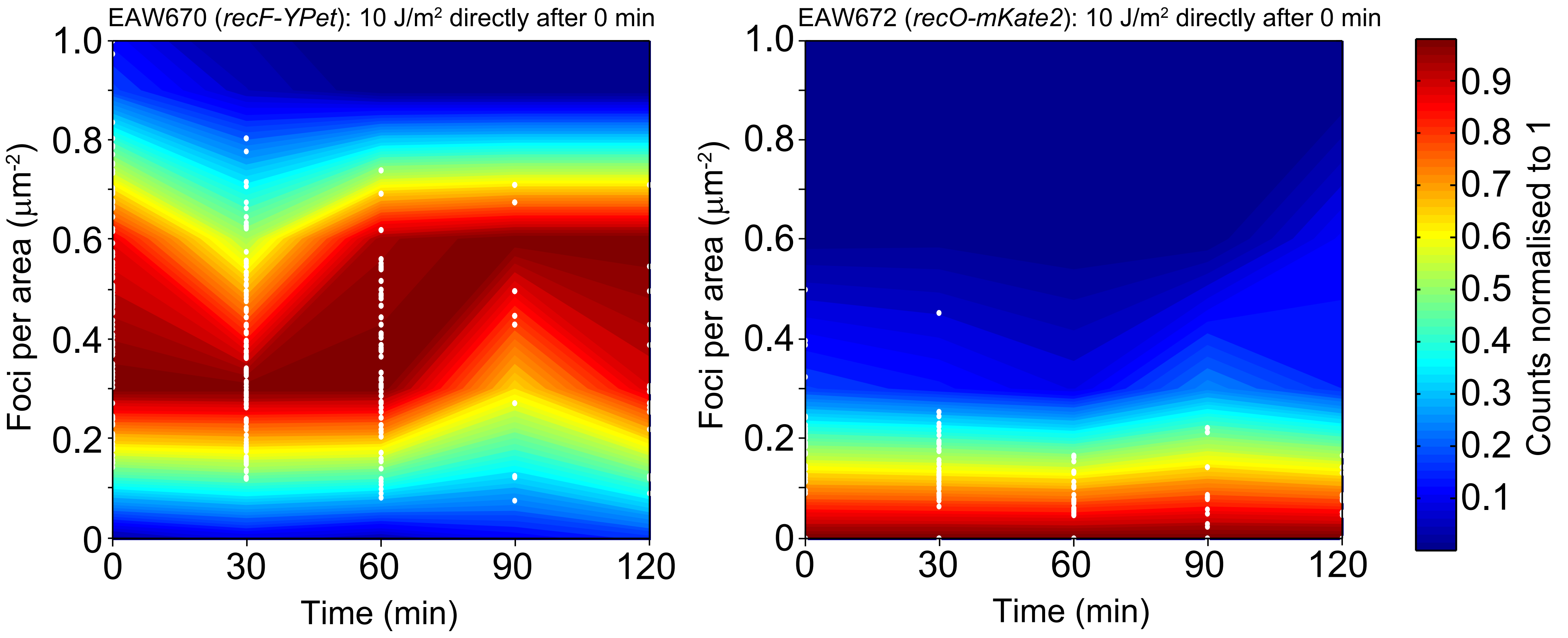

Supplementary Figure S7

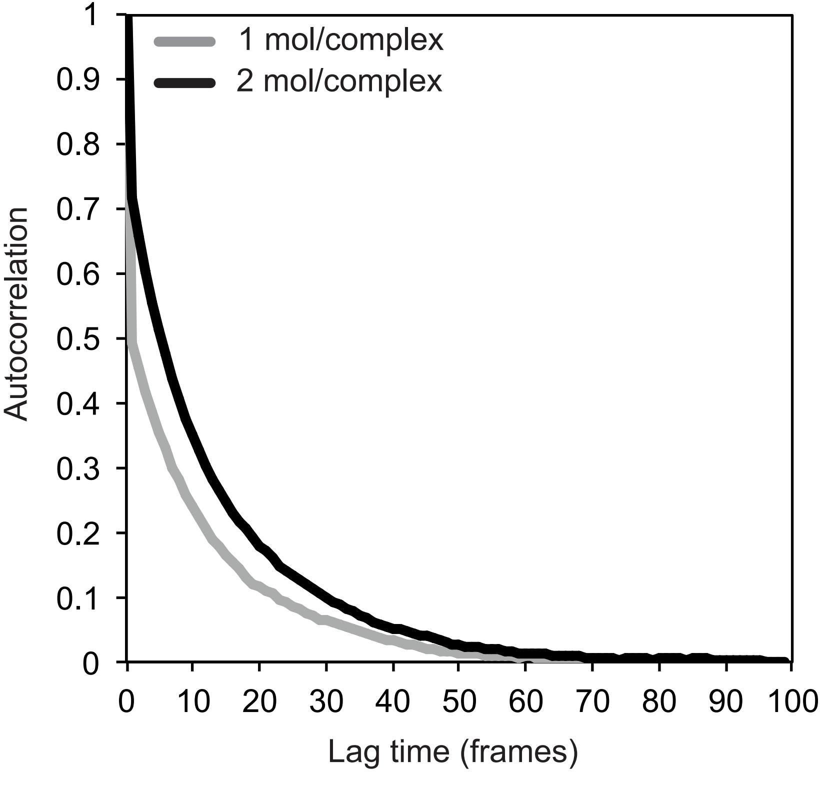

Supplementary Figure S8

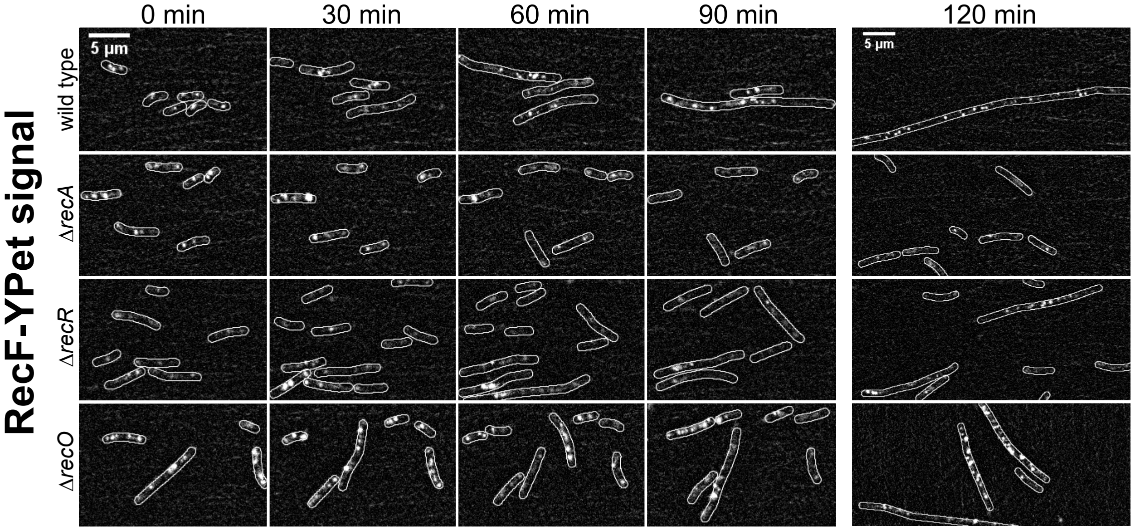

Supplementary Figure S9

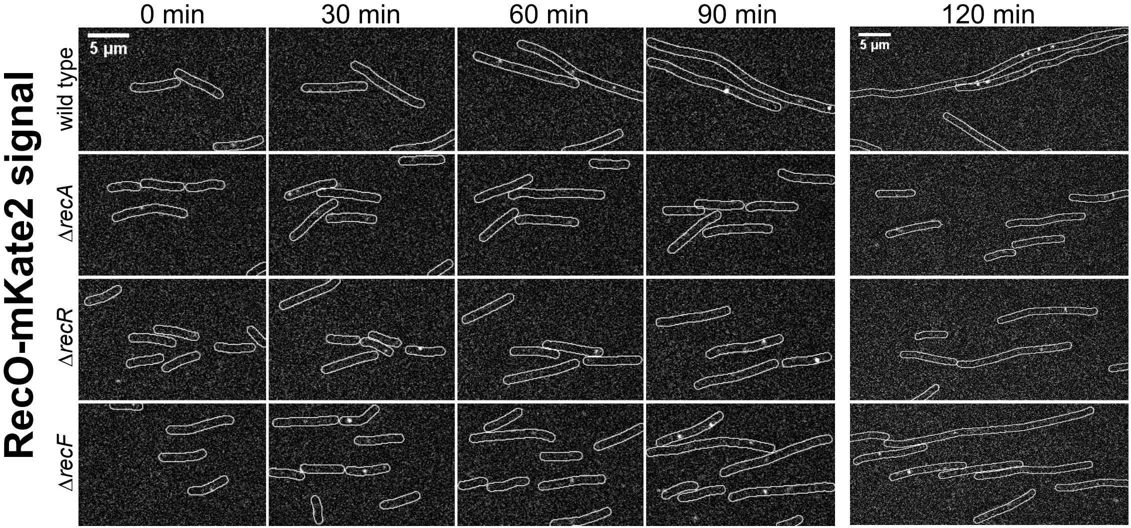

Supplementary Figure S10

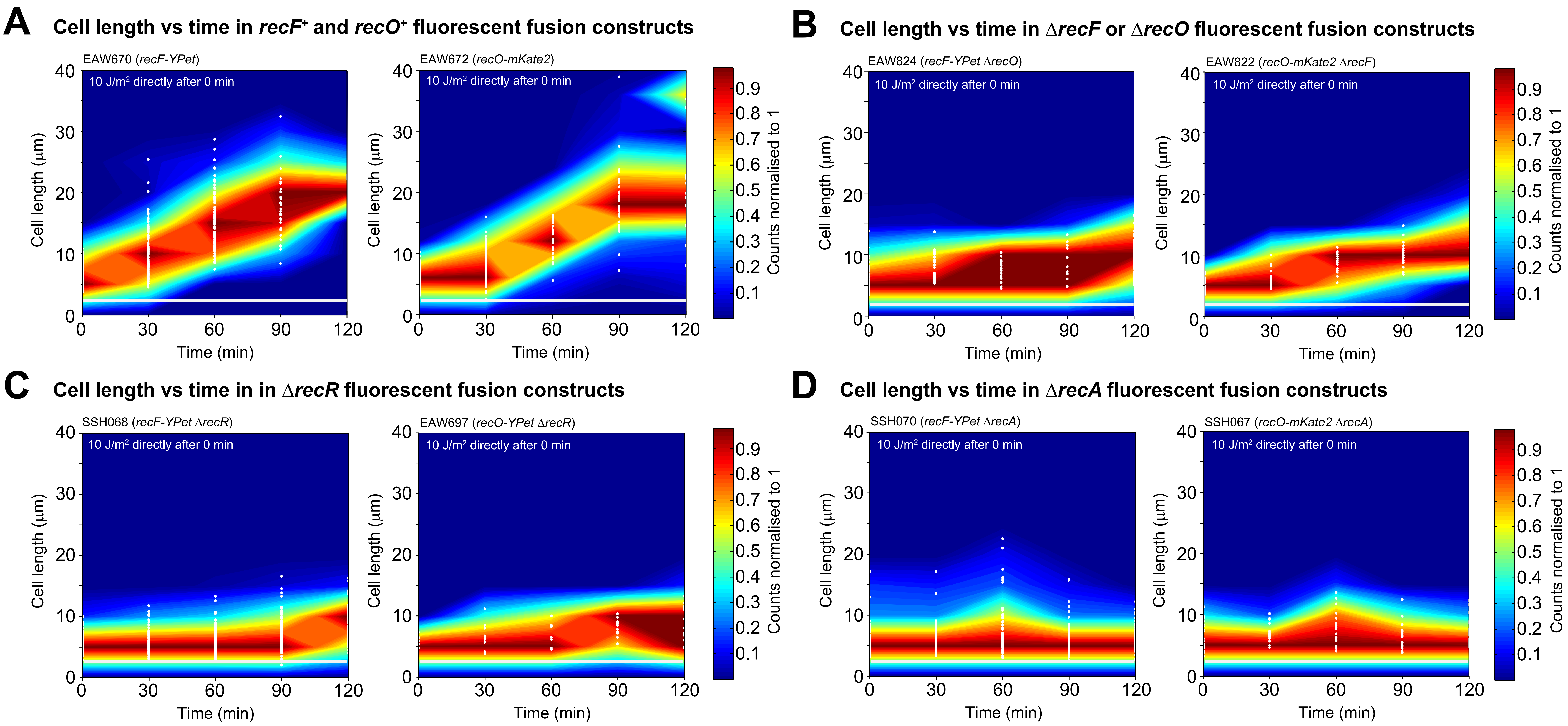

Supplementary Figure S11

Number of RecF-YPet foci per cell in *recA*, *recR* and *recO* mutants

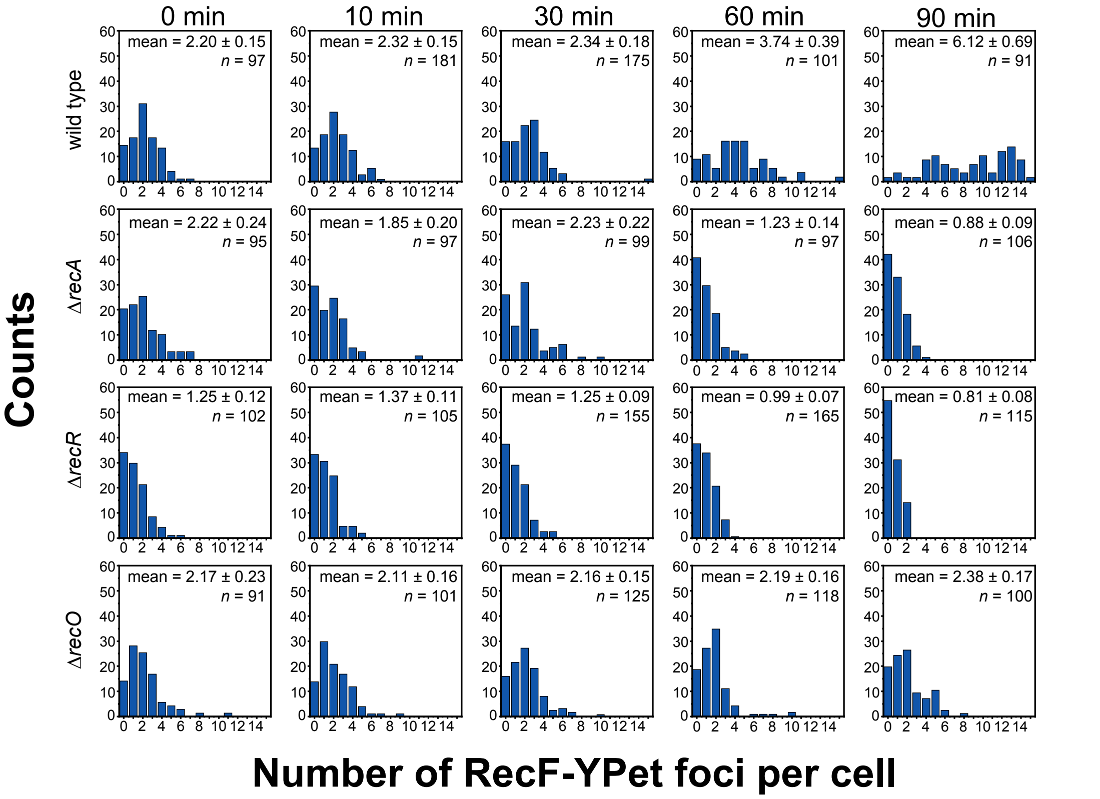

Supplementary Figure S12

Number of RecO-mKate2 foci per cell in *recA*, *recR* and *recF* mutants

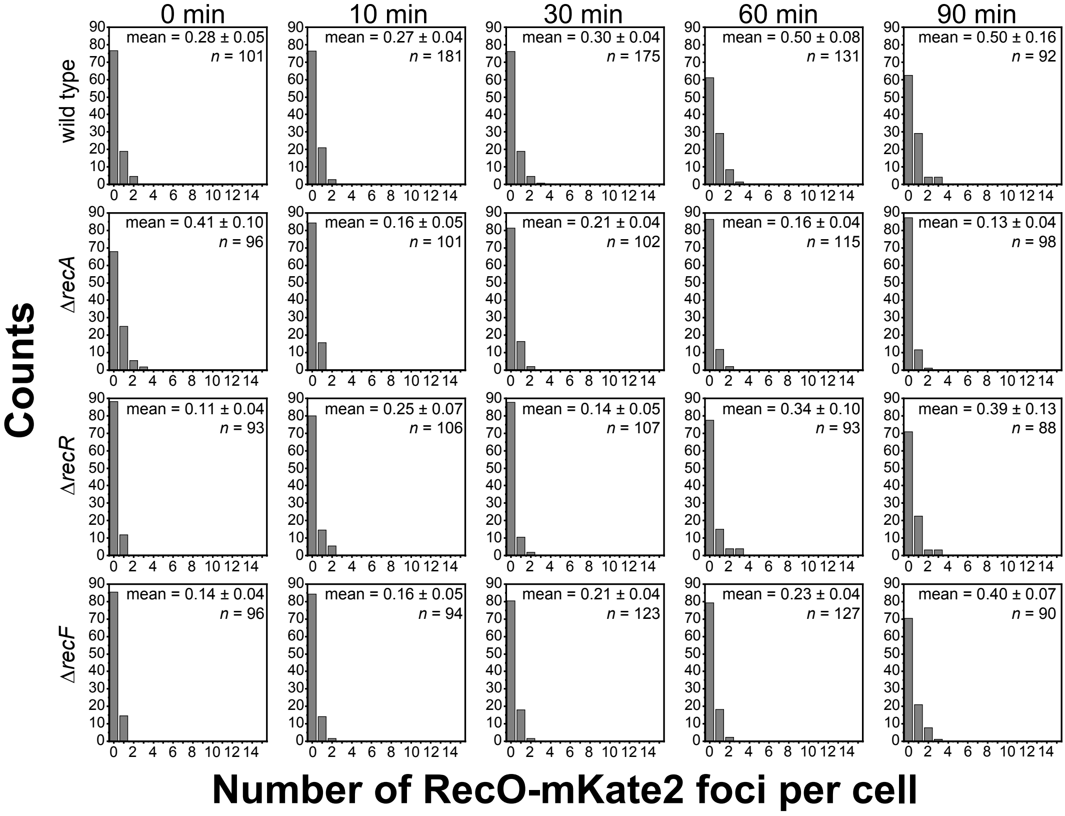

Supplementary Figure 13

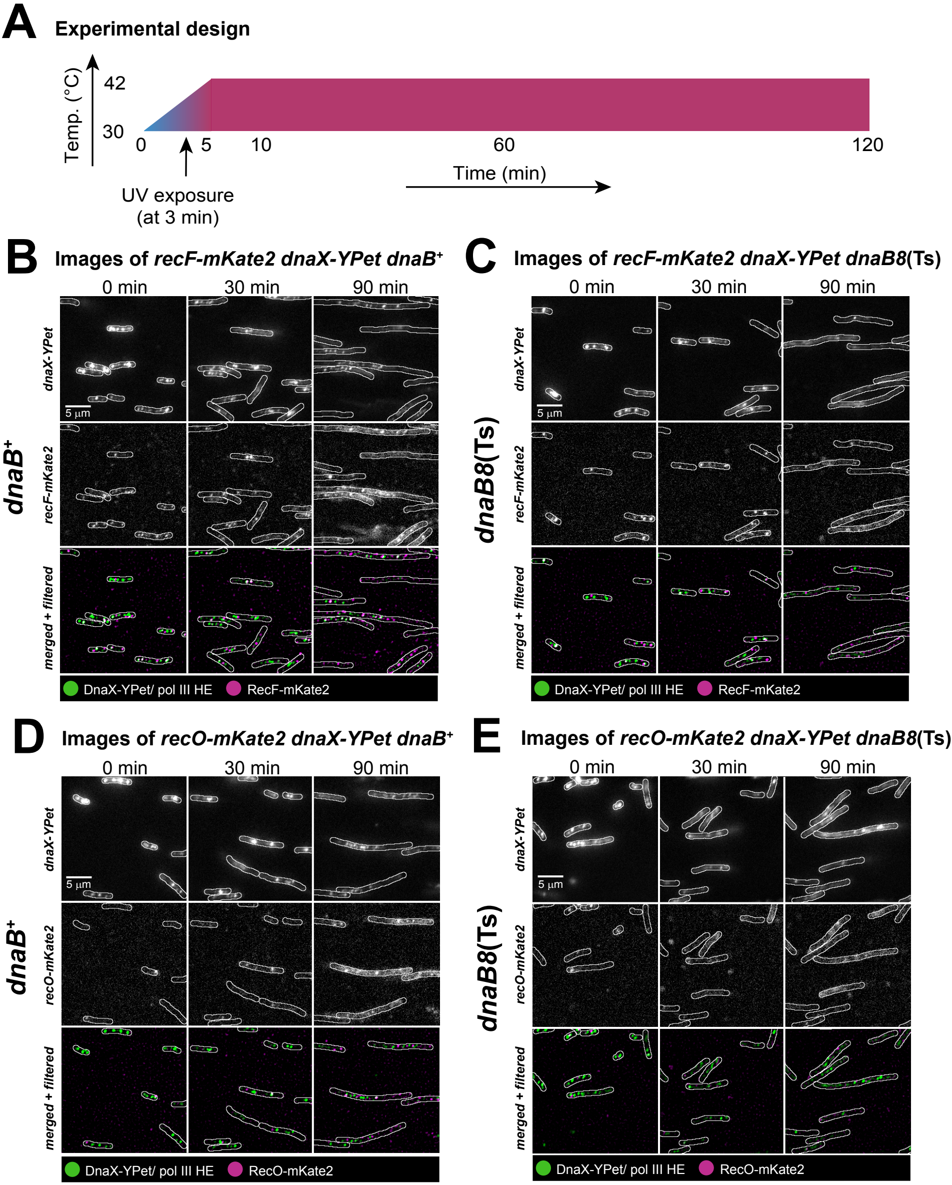

Supplementary Figure S14

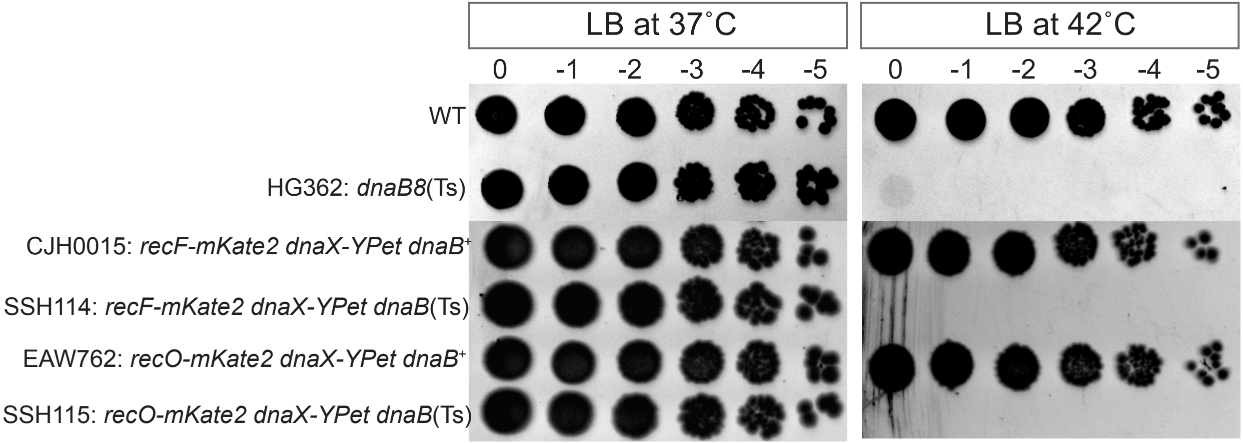

Supplementary Figure 15

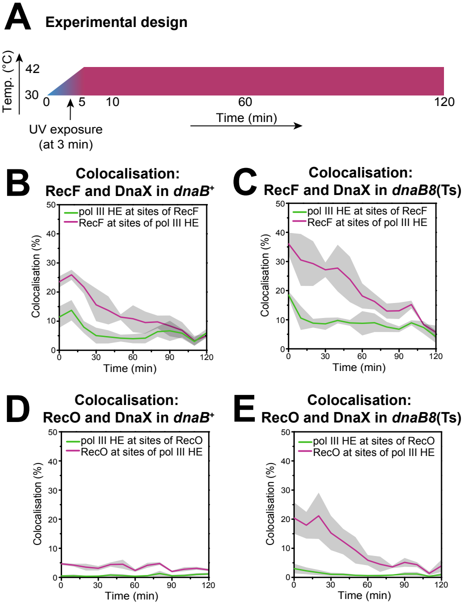

Supplementary Figure 16

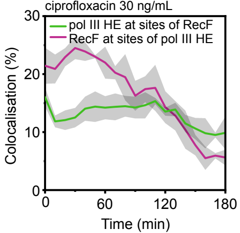

Supplementary Figure S17

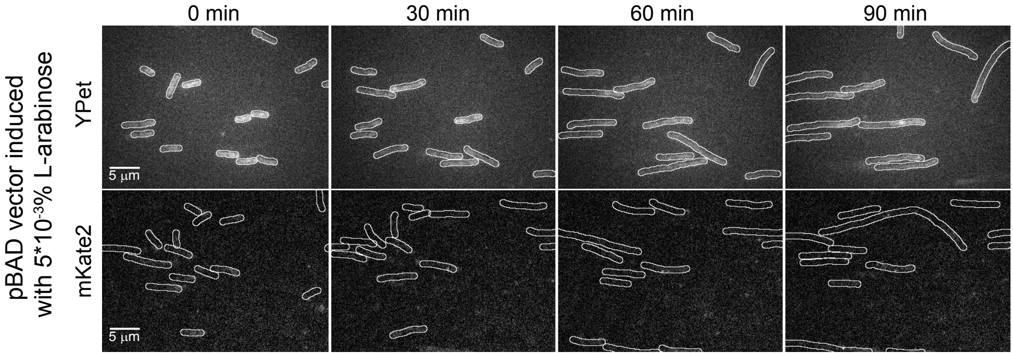

Supplement: Supplementary Data [file gkz003_supplemental_files.pdf]
